# Whole-Genome and Transcriptome Sequencing-Based Characterization of *Bacillus Cereus* NR1 From Subtropical Marine Mangrove and Its Potential Role in Sulfur Metabolism

**DOI:** 10.3389/fmicb.2022.856092

**Published:** 2022-03-09

**Authors:** Muhammad Kashif, Zhaomei Lu, Yimeng Sang, Bing Yan, Syed Jalil Shah, Sohail Khan, Muhammad Azhar Hussain, Hongzhen Tang, Chengjian Jiang

**Affiliations:** ^1^State Key Laboratory for Conservation and Utilization of Subtropical Agro-bioresources, Guangxi Research Center for Microbial and Enzyme Engineering Technology, College of Life Science and Technology, Guangxi University, Nanning, China; ^2^Key Laboratory of Bio-resources and Eco-environment of the Ministry of Education, College of Life Sciences, Sichuan University, Chengdu, China; ^3^Guangxi Key Lab of Mangrove Conservation and Utilization, Guangxi Mangrove Research Center, Guangxi Academy of Sciences, Nanning, China; ^4^MOE Key Laboratory of New Processing Technology for Non-ferrous Metals and Materials, Guangxi Key Laboratory of Processing for Non-ferrous Metals and Featured Materials, School of Chemistry and Chemical Engineering, Guangxi University, Nanning, China; ^5^Alfa Diagnostic Services, Faisalabad, Pakistan; ^6^Key Laboratory and Cultivation Base of Prevention and Treatment of Traditional Chinese Medicine on Obesity, Guangxi University of Chinese Medicine, Nanning, China

**Keywords:** *Bacillus cereus*, biodesulfurization, mangrove habitat, metabolization, molecular analysis

## Abstract

Sulfur, organosulfur compounds, and sulfides are essential parts of life. Microbial sulfate assimilation is among the most active and ancient metabolic activities in the sulfur cycle that operates in various ecosystems. We analyzed the molecular basis of bacterial characterization. NR1 was isolated and purified from mangrove sediments. Whole-genome sequencing indicated that the NR1 isolate was closely related to *Bacillus cereus*. The genome contained 5,305 functional genes with a total length of 5,420,664 bp, a GC content of 35.62%, 42 rRNA, and 107 tRNA. DBT-grown cultures exhibited DBT utilization, fleeting emergence of DBT sulfone (DBTO_2_), and formation of 2-hydroxybiphenyl (2-HBP). Molecular analysis of the PCR products’ *dsz* operon revealed the presence of *dszA, dszB*, and *dszC* genes, which encoded for NR1’s 90% DBT desulfurization activity. Furthermore, 17 sulfur metabolism-related genes, including genes involved in assimilation sulfate reduction, *APS* and *PAPS*, and the *cys, ssu*, and *TST* gene families, were identified. In sulfate media, alkenesulfonate was converted to sulfite and inhibited *ssu* enzymes. Downregulated *cysK* variants were associated with *nrnA* expression and the regulation of L-cysteine synthesis. These findings established a scientific foundation for further research and application of bacteria to mangrove rehabilitation and ecological treatment by evaluating the bacterial characterization and sulfur degradation metabolic pathway. We used whole-genome and transcriptome sequencing to examine their genetic characteristics.

**Figure:**
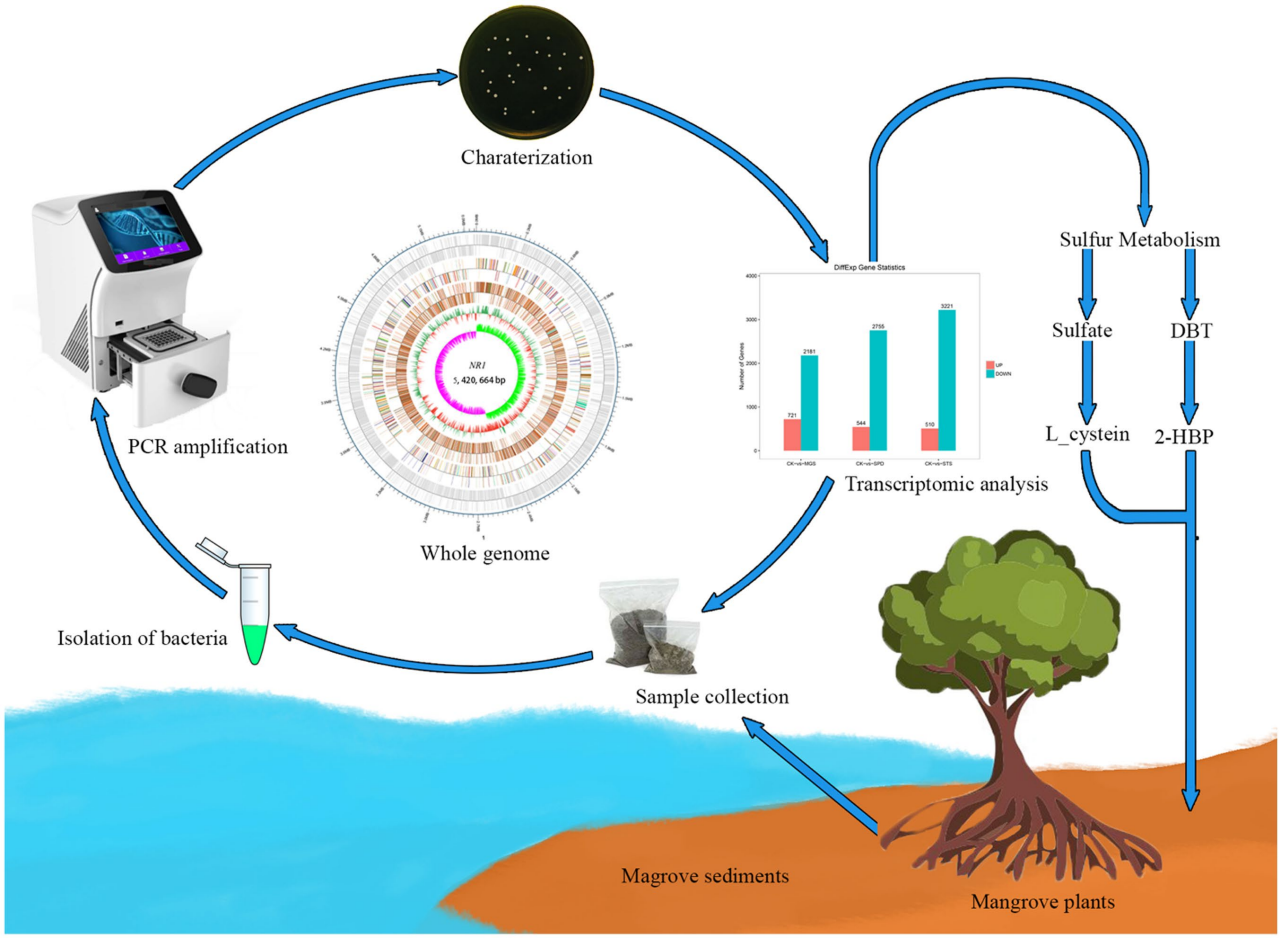
Graphical Abstract

## Introduction

Mangroves are tropical and subtropical coastal forests. The high temperature, the fluvial substrate, salty water, and the tidal range are the main determinants dictating their occurrence, structure, and functioning (Dalrymple and Choi, 2007). The inflow of sedimentary material from the sea and continents is essential to the production and evolution of these ecosystems (Buatois and Mángano, 2011). Since 2007, approximately 66 new *Actinobacteria* species and eight new genera has been isolated and characterized from mangrove habitats (Biswas et al., 2017). Bacteria are involved in various ecological cycles, including carbon, nitrogen, and sulfur; they play a role in oxidation and reduction reactions (Boyd et al., 2017).

Microorganisms have been a critical source of biotechnology products, due to their wide metabolic variety, adaptability, and relative handling on large-scale production systems. Mangroves have significant biological production, including a thriving microbial life. Usually, the vegetation and coastal outflow decrease; organic and inorganic wastes gather on a massive scale in this vital environment due to their root systems (Verma and Jaiswal, 2016). Microbes degrade most organic wastes and contribute to biological productivity either explicitly or implicitly. Heterotrophic bacteria utilize complex organic matter as an energy source, transforming it into metabolic waste products, such as water and carbon dioxide. Due to this natural process, wastes are consumed or converted into a less hazardous form. Additionally, heterotrophic bacteria have a high degree of metabolic diversity due to diverse enzymes with diverse substrate specificities (Mumford et al., 2006).

In sulfur-oxidizing bacteria (SOB), related *thiobacilli* efficiently oxidize sulfide to sulfate in the presence of sufficient oxygen without producing elemental sulfur (Ito et al., 2002). Sulfates are converted to hydrogen sulfide by sulfate-reducing bacteria (SRB) to exclude nitrates and dissolve oxygen as an additional source of oxygen to catabolize organic waste. Sulfur dioxide is a severe air pollutant with a substantial effect on human health. Furthermore, the concentration of sulfur dioxide in the atmosphere can affect habitat availability for mangrove plant communities and wildlife (Giordano et al., 2005). As a primary source of fossil fuels, sulfur is the predominant contaminant that contributing to environmental degradation and health risks through acid rain and sulfur oxide emissions (Bordoloi et al., 2014).

To minimize sulfur emissions after the establishment of rigorous pollution control standards, meeting the requirements of appropriate desulfurizing methods for large sulfur-containing fossil fuels is critical. The most common compound for sulfur degradation is dibenzothiophene (DBT; Das and Chandran, 2011). DBT has gained global attention due to its removal of organic sulfur (Mishra et al., 2016). Certain bacteria may degrade sulfur in complex organic sulfur compounds (such as DBT) for their growth and essential processes and thus can be used to remove organic sulfur from fossil fuels. Most of the bacteria oxidize sulfur in DBT *via* the 4-S pathway through enzymes such as DBT sulfone monooxygenase (*DszA*), 2-HBP desulfinase (*DszB*), DBT monooxygenase (*DszC*), and flavin reductase (*DszD*). The *dszABC* operon governs this sulfur-specific process, which does not interrupt the carbon skeleton and continues under normal physical conditions (Elmi et al., 2015).

The microbial diversity of mangrove ecosystems provides information on their ecological importance and distinct biotechnological possibilities in “omic” technologies (Dias et al., 2009). It has evolved as an innovative tool for identifying microbes and understanding their broad spectrum of biological activities in the ecosystem. To our knowledge, the study on these terms is not well reported because their suitable growth requirements cannot be replicated in laboratory or indoor environments (Sharma and Lal, 2017).

Thus, the phylogenetic analysis of bacteria and their families from mangrove sediments can better elucidate bacteria’s ecological significance in the mangrove environment. Based on the hypothesis, a bacterium from mangrove sediment would degrade sulfur and its derivatives and convert DBT to 2-HBP. Therefore, this study was conducted to evaluate the effect of certain bacteria (*B. cereus*) on biodesulfurization in mangrove sediments. We identified the bacterial strain as NR1 from *B. cereus*, and 36 genes were obtained under sulfur conditions. We also studied its vast biotechnological prospects, ecology, and genetics through whole-genome sequencing and transcriptome analysis.

## Materials and Methods

### Bacterial Strain and Processing of Sediments Collection

NR1 samples were obtained from subtropical mangrove sediments in the Beibu Gulf, South China Sea (21°29′25.74′′N, 109°45′49.43′′E), as indicated in Supplementary Figure S1, and deposited in the China Center for Type Culture Collection Center (CCTCC) under Accession Number M20211548. To prepare SOB media, we mixed the following: 0.5 g barium chloride (BaCl_2_), 0.2 g sodium bicarbonate (NaHCO_3_), 2 g potassium dihydrogen phosphate (KH_2_PO_4_), 2 g sodium thiosulfate (Na_2_S_2_O_3_), and 0.2 g calcium nitrate Ca(NO_3_)_2_. We also used 15–20 g agar per liter, 1 g yeast extract agar, and 1 g sulfur powder. The bacterial sample was made by mixing 1 g of soil sediment (NR1) with 50 ml of sterile distilled water and performing three serial dilutions (10^0^, 10^−1^, and 10^−2^) in triplicate. Then, 100 μl of each replication of the diluted sample was added into SOB medium plates. All plates were incubated for 3–4 days, and the growth of the colonies was measured. Subculturing was conducted for purification purposes and transferred to SOB broth. Approximately 100 ml of Luria–Bertani (LB) medium was cultured and incubated for 24 h at 37°C and 1 ml of sample was taken for DNA extraction. Glycerol (40% V/V) was added to the remaining cultured bacterial strain in the LB medium and culture was stored at −80°C for subsequent investigation.

### DNA Extraction, Molecular Determination, and Phylogenetic Analysis

After DNA extraction and purification, its quality was determined using electrophoresis on a 1% agarose gel and a spectrophotometer (NanoDrop 2000). For the amplification of the 16S rRNA gene, the primers 27F (5’-GAGTTTGATCCTGGCTCAG-3′) and 1492R (5′-GGTTACCTTGTTACGACTT-3′) were used under the following reaction conditions: 95°C, 5 min; [95°C for 45 S; 56°C for 45; and 72°C for 1 min] × 30 cycles; and 72°C, 10 min. The PCR products were purified with the TIANamp bacterial DNA kit (TIANJIN). Sanger sequencing (BGI)^1^ was used to sequence the purified PCR product of the 16S rRNA gene. The 16S rRNA sequence was then blasted in NCBI for pairwise comparison. NR1 as a query was used to obtain the sequences of 36 distinct bacterial strains from NCBI.^2^ MEGA 7 software was used to perform the CLUSTAL W alignment on the NR1 sequence (Kumar et al., 2018). The Maximum Likelihood technique based on the Tamura-Nei model was applied to infer the evolutionary history. The maximum log-likelihood tree (−3270.29) was identified and is shown in Supplementary Figure S2A. The taxa with the highest homology are grouped and presented alongside the branches. The Neighbor-Joining and BioNJ algorithms and a matrix of pairwise distances were computed using the maximum composite likelihood (MCL) technique. These were then utilized for systematic searches to create an initial tree(s), and the tree with the highest log-likelihood rate was chosen. The molecular genetic tree with a total of 36 nucleotide sequences was used. Missing data and gaps, including places, were deleted. Thus, 1231 positions in the final data set were obtained. All molecular phylogenetic analyses were carried out using MEGA 7 software.

### Characterization and Enrichment of Isolated Bacterial Strain

A single colony of NR1 was isolated and its morphological, biochemical, and molecular characteristics were evaluated. NR1 growth was determined using marine (2216E) and LB media. Motility, staining, growth condition, and optimum pH range varied from 5.5 to 8. Optimal temperature range, a heat resistance temperature range of 20°C–60°C, oxidase, and catalase were determined; 1% (v/v) tetramethyl-p-phenylenediamine dihydrochloride and 3% (v/v) hydrogen peroxide, as well as NaCl tolerance salt concentration from 4 to 16% were all maintained (Ray et al., 2012). Antibiotic disks containing vancomycin (30 μg), tetracycline (30 μg), cefotaxime (30 μg), streptomycin (10 μg), ciprofloxacin (10 μg), gentamicin (20 μg), cefuroxime (30 μg), cephalosporin (30 μg), penicillin G (10 μg), erythromycin (15 μg), and ampicillin sulbactam (30 μg) were used to test the antibiotic sensitivity of the NR1 strain culture (Caplan et al., 2014). The nitrogen source utilization included L-histidine, aspartic acid, L-serine, L-alanine, L-ornithine, L-hydroxyproline, D-arginine, and glycine at a final concentration of 1% (w/v). Carbon source utilization included L-arabinose, D-galactose, glucose, sucrose, D-xylose, D-fructose, D-mannitol, lactose, L-rhamnose, D-raffinose, and D-Mannose at a final concentration of 1% (w/v). β-Glucosidase, insulinase, xylanase, phytase, phosphorus, protease, degradable ammonia nitrogen, and inorganic potassium dissolution were used to test NR1 enzymatic activity (Grimont and Grimont, 2015).

### Utilization of Sulfur Source

Various sulfur media were used to culture the bacterial strain NR1, as follows: sulfur powder magnesium sulfate, sodium thiosulfate, sodium sulfide, and sodium sulfite. Because of the varying oxidation states of sulfur in the medium and their relationship with the enzymatic reactions of NR1. Except for a change in the sulfur source, the composition of bacteria growth media was the same. The medium consisted 1 g ammonium chloride (NH_4_Cl), 2 g sodium bicarbonate (NaHCO_3_), 2 g monopotassium phosphate (KH_2_PO_4_), 0.8 g magnesium chloride (MgCl_2_), 1 g potassium nitrate (KNO_3_), and 5 g of a single sulfur source which are sulfur powder (99.5% pure), magnesium sulfate (MgSO_4_), sodium thiosulfate (Na_2_S_2_O_3_), sodium sulfite (Na_2_SO_3_), and sodium sulfide (Na_2_S) to make the final volume to 1 l (Kantachote et al., 2008).

### Biodesulfurization Process

In 50 ml deionized water, 2.7 mmol of DBT and 200 μl dimethylformamide (DMF) were mixed. Then, 0.125 g of NR1 (bacterial strain) was added, and a sample was agitated at 37°C and 200 rpm in an incubator. The oil (n-octane) phase of the solvent was extracted using a separatory funnel after 25 h. A UV–Visible spectrophotometer (Shimadzu, Japan) was used to measure the remaining DBT in the oil phase at 325 nm (Shah et al., 2016).

### Microbial Sulfur Metabolic Pathway

KEGG annotations were used in the KEGG Automatic Annotation Server using the BBH-method (KAAS). The complete NR1 genome was automatically annotated. The sulfur cycle for NR1 and the annotated results were compared using KEGG Automatic Annotation Server (KAAS) through BLAST^3^ according to the method of Moriya et al. (2007).

### Whole-Genome Assembly and Annotation

The whole-genome sequencing was performed by PFOMIC Bioinformatics Company in Nanning, China, using the Illumina NovaSeq 6000 PE150, Oxford Nanopore Technologies PromethION. The extracted DNA samples’ quality, purity, concentration, and integrity were determined using the following parameters: (i) appearance (whether the sample contains any defilement); (ii) degradation and DNA fragment size (as determined by agarose gel electrophoresis); (iii) DNA purity (as determined by nanodrop); and (iv) 200 ng concentration (Qubit, set as a criterion for accurate DNA quantification). After qualifying the sample, an EXP-NBD104/114 kit was used to add a barcode label, and magnetic beads were used to screen for target DNA fragments. The purified product was linked to a sequencing connector and the sqK-LSK109 kit, and Qubit was used to quantify the constructed DNA library (Creason et al., 2021). After passing the test, the DNA samples were randomly interrupted using a Covaris ultrasonic crushing instrument. The entire library was prepared using the following procedures: terminal repair, the addition of an A tail, the addition of a sequencing connector, purification, and PCR amplification. After constructing the library, Qubit 2.0 was used to perform preliminary quantification and dilution. Agilent 2,100 was used to detect the library fragments (Jain et al., 2016). Then, the effective concentration of the library was quantified using the qPCR method. The cBOT was clustered and sequenced using the Illumina high-throughput sequencing platform NovaSeq 6000. The low-quality readings were filtered using the MinKNOW software, which used Pass reads (*Q* > 7) as a criterion. To assemble the filtered reads, Unicycler (0.4.8) was utilized (Wick et al., 2017). To acquire high-quality bacterial genome contigs, the contig was first assembled with highly accurate Illumina data (*Q*_30_ > 85%). Afterward, the high-quality contigs were linked to the finalized graph using Nanopore data. The correctness of Illumina data dictated the bacterial completion graph created, which effectively mitigated sequence pollution caused by nanopore data separation inaccuracy (Chen et al., 2020).

Finally, the assembled genome was corrected with second-generation data using Pilon software, resulting in a more accurate final genome. Prokka software was used to predict the assembled genome (Seemann, 2014). Prokka (Prodigal), a set of genetic component prediction tools, was used to predict encoding genes; Aragorn was used to determine Barrnap/Rnammer and tRNA. Eventually, all RNAs were integrated and annotated. To estimate gene functions, whole-genome alignment (E-value 1e-5 and a minimum alignment width percentage >40%) was performed against multiple databases, including Gene Ontology (GO), Kyoto Encyclopedia of Genes and Genomes (KEGG), Non-redundant (Nr), Pfam, and Swiss-Prot (Bairoch and Apweiler, 2000). The VFDB and ARDB databases were used to investigate pathogenicity and drug resistance fields (Liu et al., 2019). Moreover, the Circos software analyzed the genome sample and coded gene prediction (Zhang et al., 2013). Genome GC content, GO, KEGG gene function annotation, and genome GC skew value distribution were used to identify genome samples (Botstein et al., 2000; Kanehisa et al., 2004; Alcock et al., 2020).

### Library Preparation and Sequencing for Transcriptomic Analysis

The TRIzol-based technique was used to extract total RNA (Life Technologies, CA, United States). The NanoPhotometer^®^ spectrophotometer was used to verify RNA purity (OD_260_/OD_280_; OD_260_/OD_230_; IMPLEN, CA, United States). ProtoScript Reverse Transcriptase (New England BioLabs, Ipswich, MA, United States) was used to synthesize primary stranded cDNA using the Purification Kit (Bacteria; Illumina, San Diego, CA, United States). Bioanalyzer 2100 was used to analyze RNA integrity (Agilent, Santa Clara, CA). The Illumina MRZB12424 Ribo-Zero rRNA was used to eradicate rRNA from 1 μl of total RNA. The secondary stranded cDNA was prepared with buffer and a combination of dUTP, dGTP, dATP, and dCTP. The USER enzyme mix was then used to disintegrate the second-strand cDNA (New England BioLabs, Ipwich, MA, United States). Finally, the raw reads were produced and sequenced on the Illumina Novaseq 6000 platform (GENE DENOVO, China; Ju et al., 2019).

### Real-Time PCR Analysis

The transcriptome data were validated using real-time quantitative PCR. For this, total RNA was isolated using the Trizol technique. According to the manufacturer’s instructions, the isolated RNA was transcribed to cDNA using a reverse transcription kit (6210A TaKaRa, Japan). The PCR cycling profile was carried out under the following conditions: 5 min at 95°C, followed by 22 cycles of 30 s at 94°C, 30 s at 57°C and 30 s at 72°C, with a final extension of 10 min for 72°C. The 2^△△Ct^ procedure was applied to evaluate the qRT-PCR data. The experiment was performed in triplicate. β-Actin was used as the reference gene for the normalization (Livak and Schmittgen, 2001).

## Results

### Molecular Phylogeny

The strain NR1 had the closest phylogenetic relationship to *B. cereus* according to the taxonomy identification performed with MEGA7. A 16S rRNA-based Neighbor-Joining phylogeny with 36 other known *Bacillus* species was generated to comprehend a better evolutionary relationship of NR1 strains with other *Bacilli. B. cereus* belongs to the group of *Baciliacea* species. The precise evolutionary relationship of *B. cereus* was discovered by phylogenetic analysis. NR1 was shown to be related to *cereus* bacterial species (Supplementary Figure S2A).

### Screening and Characteristics of the Isolated Bacterium

The physiological characterization of NR1 revealed that the cells are Gram-positive, spore-forming, and motile rods. NR1 colonies were milky white, granular, and ranged in size from 1 to 6 μm (Supplementary Figure S2B). The strain NR1 grew moderately on Luria–Bertani and 1622E media (Supplementary Figure S3). Isolates grew effectively at pH levels ranging from 5.5 to 8. Optimum growth was observed at pH 7 (Supplementary Figure S4). Moreover, the optimum growth curve was observed at 17 h (Supplementary Figure S5). In Marine Broth 2216, the optimal heat resistance range was tested at a temperature range of 20–40°C, mostly 35°C (Supplementary Figure S6). The best growth of NR1 was obtained in 4% sodium chloride (Supplementary Figure S7). The catalase and oxidase tests had positive results with intervals of 3% (v/v) hydrogen peroxide and 1% (v/v) tetramethyl-p-phenylenediamine dihydrochloride (Supplementary Figure S8). NR1 strain was susceptible to the antibiotic disks, i.e., Vancomycin (30 μg), Tetracycline (30 μg), Cefotaxime (30 μg), Streptomycin (10 μg), Gentamicin (20 μg), Cefuroxime (30 μg), Cephalosporin (30 μg), Penicillin G (10 μg), Ciprofloxacin (10 μg), Erythromycin (15 μg), and Ampicillin sulbactam (30 μg; Supplementary Figure S9). Furthermore, after incubation at 37°C, nitrogen as the sole nitrogen source at a final concentration of 1% (w/v) was investigated on a 2216E Marin medium. A discrepancy in nitrogen source consumption was found, e.g., D-Arginine was highly utilized by NR1. Moreover, NR1 positively consumed aspartic acid, L-Hydroxyproline, and L-ornithine. NR1 used L-Histidine, L-serine, L-Alanine, Glycine, and L-Histidine as weak nitrogen sources (Supplementary Figure S10). NR1 reacted similarly to the carbon source, which demonstrated a strong positive utilization of a single carbon source and five positive and three weak responses to carbon source utilization. NR1 strongly utilized sucrose and other sources. Glucose, D-Galactose, D-Mannitol, and Lactose were positively utilized, whereas D-Xylose, D-Fructose, and L-Arabinose were weak in terms of source utilization (Supplementary Figure S11). Enzymatic media, such as insulinase, xylanase, phytase, protease, ammonia nitrogen, and potassium, exhibited negative growth interaction. However, β-Glycosidase exhibited high enzyme production. Phosphorous showed weak interaction with NR1 (Supplementary Figure S12; Table 1).

**Table 1:** Physicochemical characterization of NR1 bacterial strain.

**Morphological characteristics**		
Cell size		1–4 μm
Shape		Long rod-shaped
Spore formation	+
Motility		+
Gram staining		+
Growth condition		+
Color of colony		Milky sticky White
Growth on/at: Optimum pH		+ 5.5–8 (7)
Optimum temperature		17 h
Range		20–40°C
Heat resistance		37°C
**Physiological characteristics**		
Oxidase test		+
Catalase test		+
NaCl tolerance(optimum) (%, w/v)		0–16(3)
**Antibiotic sensitivity test**		
Vancomycin (30 μg) Tetracycline (30 μg) Cefotaxime (30 μg) Streptomycin (10 μg) Ciprofloxacin (10 μg) Gentamicin (20 μg) Cefuroxime (30 μg) Cephalosporin (30 μg) Penicillin G (10 μg) Ciprofloxacin (10 μg) Erythromycin (15 μg) Ampicillin sulbactam (30 μg)		S S S S S S S S S S S S
**Nitrogen source Utilization**		
L-Histidine		W
Aspartic acid		+
L-Serine		W
L-Alanine		W
L-Ornithine		+
L-Hydroxyproline		+
D-Arginine		Strongly positive
Glycine		W
**Carbon source utilization**		
L-Arabinose		W
D-Galactose		+
Glucose		+
Sucrose		Strongly positive
D-Xylose		W
D-Fructose		W
D-Mannitol		+
Lactose L-Rhamnose D-Raffinose D-Mannose		+ W + W
**Enzyme activities**		
β-Glycosidase		+
Insulinase		−
Xylanase		−
Phytase		−
Phosphorous		W+
Protease		−
Ammonia nitrogen		−
Potassium		−

*The biochemical and morphological characteristics of NR1; +, −, and W^+^ show that the responses to a specific parameter were positive, negative, and weak positive, respectively*.

### Sulfur Source Utilization

The isolated strain NR1 grew best in magnesium sulfate and sulfur powder media and grew slowly on sodium thiosulfate, sodium sulfide, and sodium sulfite media. Such findings were due to sulfur salts’ distinct natures and their interaction with the isolate’s enzymatic activity.

**Figure 1:**
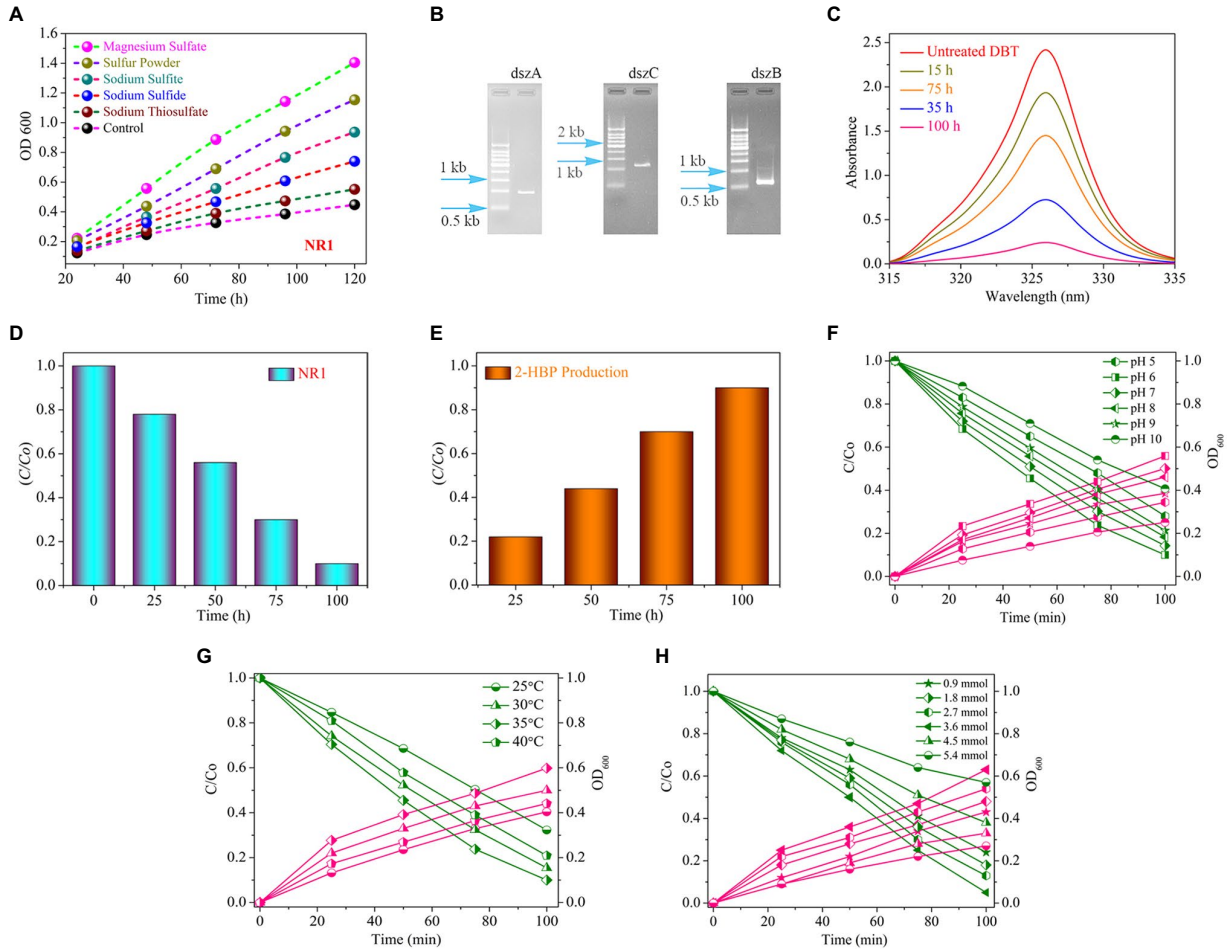
**(A)** The growth curve of the bacterial strain NR1 in various sulfur media in different time and incubation periods at 37°C (magnesium sulfate, sulfur powder, sodium thiosulfate, sodium sulfide, and sodium sulfite). **(B)** The PCR findings indicated DNA fragment imaging; NR1 intended to amplify the *dszA* (0.54 kb), *dszB* (0.42 kb), and *dszC* (1.25 kb) genes, which are implicated in the desulfurization of DBT via the 4S pathway. **(C)** The UV–Vis spectrum of DBT, **(D)** The histogram indicated DBT degradation, **(E)** Production of 2-HBP at different intervals of time in the presence of NR1 at 37°C and pH 7, **(F)** The effect of pH (5–10), **(G)** The effect of temperature (25°C–40°C), and **(H)** The initial DBT concentration (0.9–5.4 mmol) on DBT biodegradation and bacterial growth over NR1 at different time intervals (0–100 h) and 37°C.

Sodium sulfite, sulfur powder, sodium thiosulfate, and sodium sulfide are electron donors for the growth of NR1. In comparison, sulfite and sulfide media were not suited for the proper growth of the NR1 strain due to the distinct nature of sulfur bound to other elements. As a result, no growth was observed in these media. The NR1 isolate did not grow significantly in the control medium due to the lack of reduced species (electron-donating substances; Figure 1A; Ito et al., 2004).

### Biodesulfurization Process

The absorption peak and the incubation period of NR1 had an inverse correlation (Figure 1C). NR1 showed markedly excellent biodegradation activity for desulfurizing DBT (2.7 mmol). At 100 h time intervals, approximately 90% DBT was biodegraded (Figure 1D). The presence of the *DszD* gene family, which includes *dszA*, *dszB*, and *dszC* genes, was responsible for NR1’s significant biodegradation-related activity (Figure 1B). The enhanced NR1 bacterial isolate used the 4S pathway to desulfurize DBT. The *DszC* gene was related to two sequential DBT oxidation reactions and converted to DBTO (DBT-sulfoxide) and subsequently to DBTO_2_ in the DBT pathway. In contrast, all of the NR1 *DszD* family genes were involved in the enzymatic catalytic reactions of the DBT pathway (DBT sulfone). The C-S bond in DBTO_2_ was cleaved by *DszA*, resulting in HBPS. Furthermore, the *DszB* gene was implicated in the conversion of HBPS to 2-HBP, and sulfite was the end product (Figure 1E). FMNH_2_ was utilized as a reducing agent by *DszD* gene family and involved in the oxido-reduction of DBT to 2-HBP (Supplementary Figure S13). The biodesulfurization activity of NR1 was tested at pH levels ranging from 5 to 10. (Figure 1F). pH 6 was highly noticeable, along with the apparent rate constant of 15.1 × 10^3^ h^1^ (Supplementary Figure S14A). Additionally, the effect of temperature on NR1 biodegradation efficiency revealed that at 35°C, more excellent degradation kinetics was observed (Figure 1G), with an apparent rate constant of 14.0 x 10^3^h^1^ (Supplementary Figure S14B). Similarly, at 3.6 mmol of DBT concentration (15.3 × 10^3^ h^1^), higher DBT degradation rates were observed (Figure 1H and Supplementary Figure S14C). With increasing DBT concentrations, the apparent rate constant values decreased (Figure 1).

### Sulfur Metabolic Regulation Pathway

The whole-genome sequence of NR1was annotated using the KEGG, Nr, and GO databases. A total of 65 genes were identified, and these were associated with the energy metabolism pathway and then combined with the SMDB database for functional gene comparison. Seventeen genes were related to sulfur metabolism. A relatively complete assimilated sulfate reduction pathway and the cysteine biosynthesis pathway were found through whole-genome sequencing.

**Figure 2:**
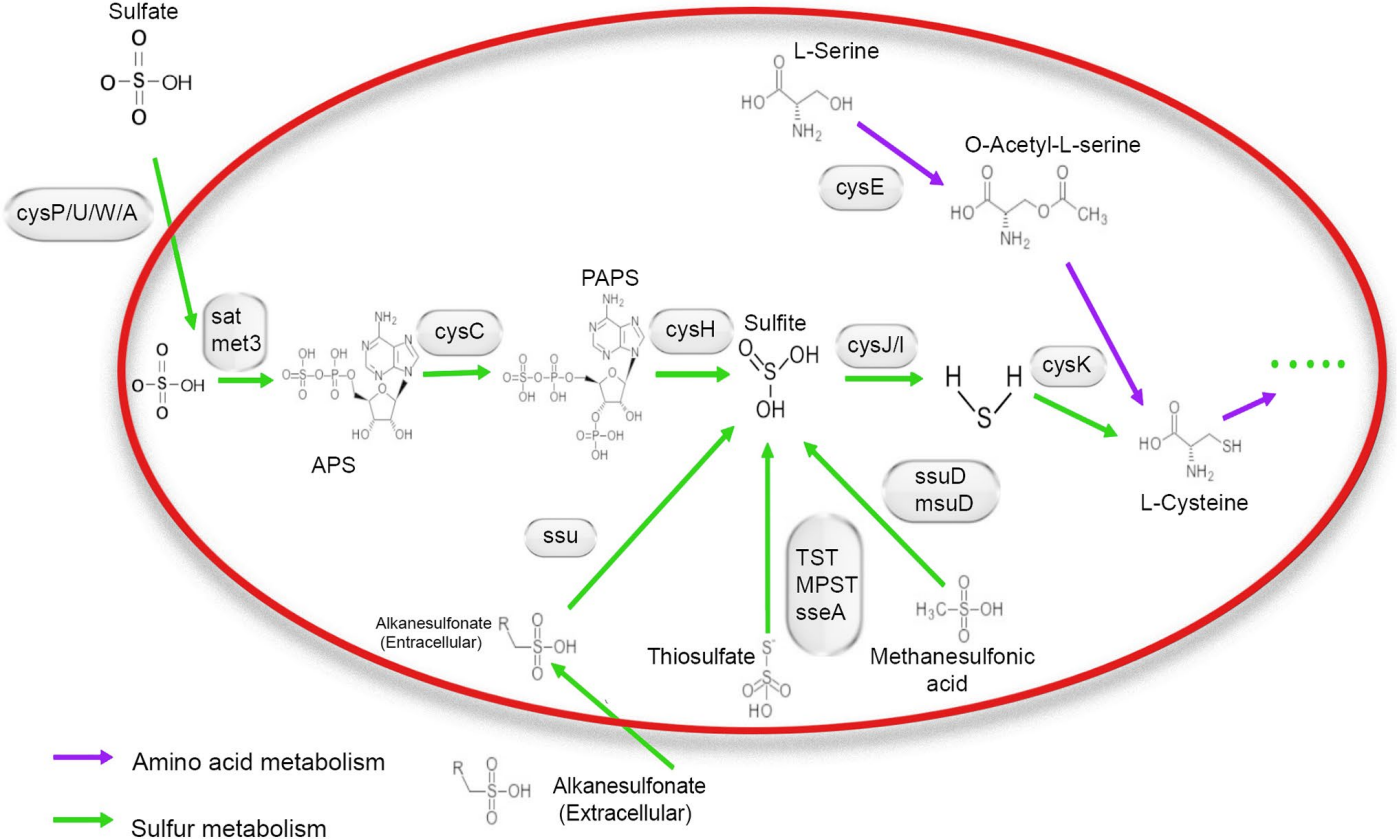
Mechanism of sulfur metabolism-related pathway and gene involved in different steps. The green arrow shows amino acid metabolism, and the blue arrow indicates sulfur metabolism.

Based on the annotation information of the whole genome, all genes were required for the assimilatory sulfate reduction pathway in the NR1 genome. This finding indicated that the sulfate reduction pathway in *Bacillus* is an assimilation pathway (Yu et al., 2018), as NR1 carrying out assimilation sulfate reduction. The genes of the sulfate/thiosulfate transport system are composed of *cysP, cysU*, *cysW*, and *cysA* (Lyubetsky et al., 2013). Therefore, it is predicted that NR1 can transport sulfate into the cell *in vitro* and generate thiocyanate through all pathways of assimilation sulfate reduction; it can also enter amino acid metabolism through cysteine biosynthesis (Kawano et al., 2018). In NR1, this sulfur metabolism pathway may be widely present in *Bacillus* species and is capable of sulfur metabolism, thereby predicting the high sulfur utilization capacity (Figure 2).

### Whole-Genome Assembly and Annotation

NR1 was annotated through the GO database, and a total of 15,855 genes were annotated. A total of 3,518 genes were related to cellular components, accounting for 22.18% of the total genes; 7,947 genes were related to biological process, accounting for 50.12% of the total genes; and 4,390 genes were related to molecular function, accounting for 27.68% of the total genes. The molecular function annotation primary gene of NR1 was related to catalytic activity and cell part. The highest numbers of genes were 1911 for the metabolic process and 1932 for the cellular function. The specific annotation results are shown in Supplementary Figure S15.

After comparison with the KEGG database, 1,436 genes in NR1 were annotated as part of 25 metabolic pathways, and the relevant genes and their metabolic pathways were also identified. A total of 222 genes were associated with environmental processing information and were involved in two metabolic pathways; 183 genes were related to genetic processing information and were involved in three metabolic pathways. However, there are 38 genes related to cellular processes and are involved in three metabolic pathways. Nineteen genes were associated with human diseases and involved in three metabolic pathways. A total of 971 genes were associated with metabolism with 11 metabolic pathways. In these 11 metabolic pathways, 290 genes were associated with amino acid metabolism, 7 genes involved in the production of secondary metabolites, 21 genes in polysaccharide and lipid biosynthesis, 210 genes in carbohydrate metabolism, 65 genes in energy metabolism, 77 genes in the lipid metabolism, 119 genes in cofactor and vitamin metabolism, 3 genes in organic systems, 26 genes in exogenous biodegradation, 25 genes in trpenoids and polyketides metabolism, 101 genes in nucleotide metabolism, and 30 genes were associated in other amino acids metabolism (Supplementary Figure S16).

In addition, the entire genome of NR1 was annotated in the Nr database. By categorizing the comparable species and the number of genes associated with each, we found that 4,551 NR1 genes were annotated to *B. cereus*, accounting for 63.84% of all annotated genes. The results of the specific annotations are as follows (Supplementary Figure S17).

**Figure 3:**
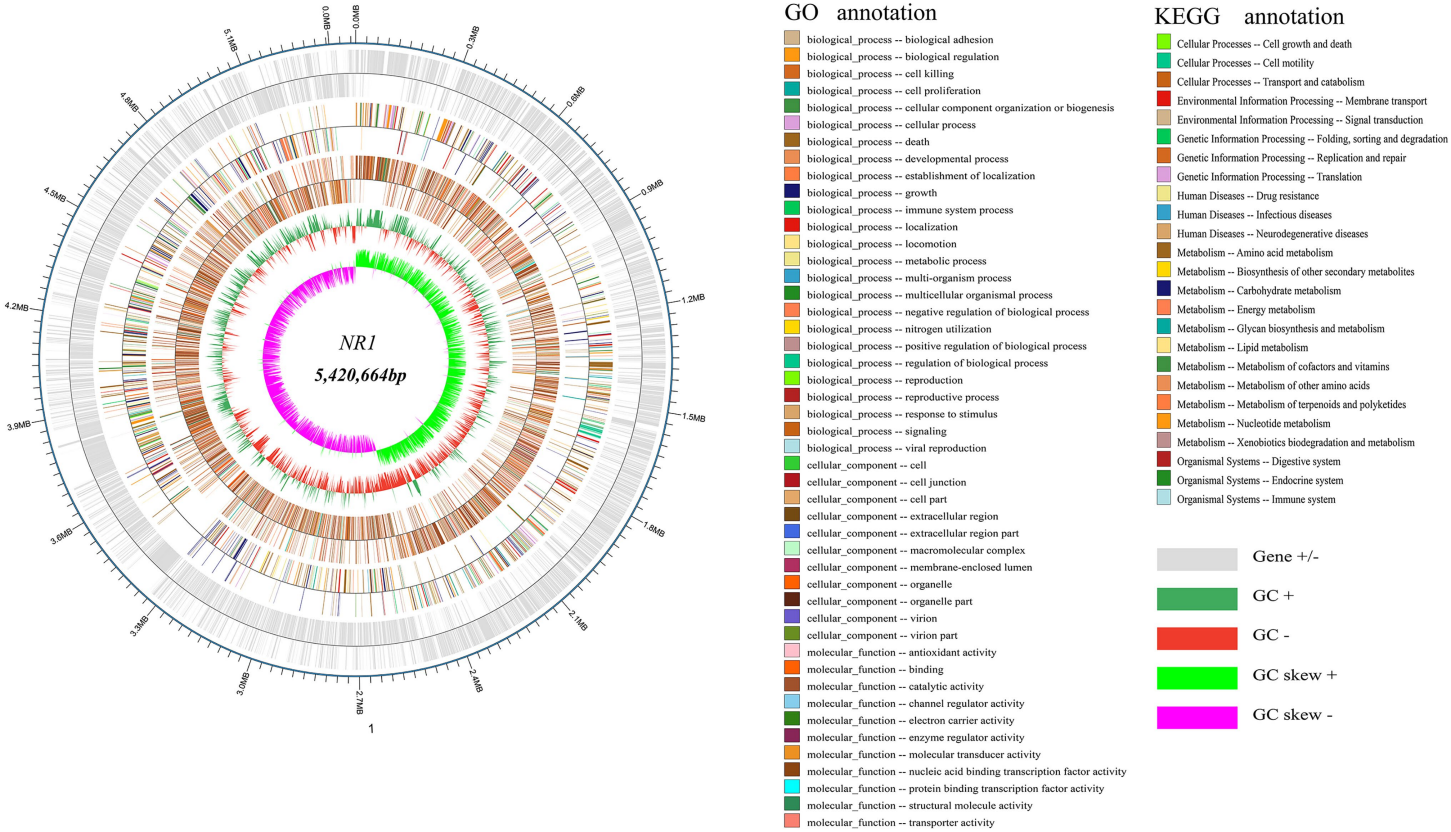
NR1 genome-wide mapping. The dark and light silver circles denoted genes (+ and -), the red and dark green circles represented GC content, and the purple and light green circles showed the GC skew. The first innermost circle represented genome GC skew values, the second circle described genome GC content, the third circle showed Nr annotation, the fourth circle displayed GO function annotation, and the fifth circle expressed KEGG function annotation. The outer circle depicted the result of the coding gene annotation.

The whole genome of NR1 is shown in Figure 3. Supplementary Table S1 summarizes the basic properties of the NR1 genome. The circular chromosome of NR1 was 5,420,664 bp in size. Aragorn was used to predict tRNA, Prodigal tools were used to predict encoding genes, and all of these were finally aggregated and annotated (Supplementary Table S1). We sequenced and assembled the *de novo* genome using Illumina and Nanopore technologies to obtain an accurate map of the NR1 genome and performed in-depth bioinformatics tools in this project. The genome was 8.11 Mb in size with a GC content of 35.62%.

About 5,305 functional genes were predicted for NR1, of which 107 were related to tRNAs, 125 to ncRNAs, and 42 to rRNA. The functional KEGG database annotated 1,436 genes belonging to 25 functional metabolic pathways, and the GO database annotated 15,855 genes. In contrast, the Nr database annotated 4,551 genes of the NR1 complement *Bacillus* (Figure 3).

### Transcriptome Sequencing and DEGs Analysis

The qualities of sulfur powder (SPS), magnesium sulfate (MGS), sodium thiosulfate (STS), and control (CK) groups’ transcriptomic data were determined using Phred quality rates (Q), which were logarithmically correlated with the percentages of base inaccuracy (P). The resulting clean data received a minimum Q score of 92.01% (Q_30_). The proportion of alignment for clean data mapped to the reference genome was 94.89 to 99.45%, whereas the percentage of multiple mapped reads was 0.78 to 4.02%. Moreover, the clean read, GC content, and clean bases ranged from 8,211,394 to 10,342,584, 42.22 to 46.98%, and 1,158,392,988 to 1,525,678,120, respectively (Supplementary Table S2).

The annotated mapped reads with the reference genome were linked to innotate the assembly. After filtering the sequences, the SPD, MGS, and STS transcripts were blasted against the reference genome for transcript annotation, revealing 5,305 genes in each group. All genes were run through various databases, including KEGG, GO, Swiss-Prot, and Nr. The number of transcripts annotated in different databases in each category was determined.

Differential analysis was performed to identify the gene clusters involved in sulfur degradation. NR1 showed thriving growth in the sulfur medium and poor growth in the non-sulfur sources, and these results were obtained by estimating physicochemical characteristics. According to the transcriptome results, differences existed in a significant number of DEGs of SPD, MGS, and STS groups (3,299, 2,902, and 3,731), respectively. To determine differentially expressed genes (DEGs), EdgeR software was used, and log2FC ≥ 1 and *p* < 0.05 were the testing parameters. Similar gene expression patterns were clustered together. The findings of the DEG clustering study for the three groups are shown in supplementary table (Supplementary Table S3).

The DEGs from the groups, CK versus SPD, and CK versus MGS, and CK versus STS, were searched in the enrichment analysis of the KEGG pathway. The differentially expressed genes were associated with sulfur and nitrogen metabolism, and in the degradation of photosynthesis, valine, leucine, and isoleucine (Supplementary Figure S18).

Furthermore, the DEGs from the sulfur groups were analyzed for functional annotation in the GO database biological process, cellular component, and molecular function. The biological processes related to DEGs were biological regulation, cellular process, localization, metabolic process, single-organism processes, and response in all the groups. The cellular components related to these groups were the macromolecular complex, cell membrane, and cell parts. The DEGs of the three sulfur groups associated with the molecular function were involved in transporter activity, transportation factor protein binding, nucleic acid binding transportation factor, and catalytic activity (Supplementary Figure S19).

**Figure 4:**
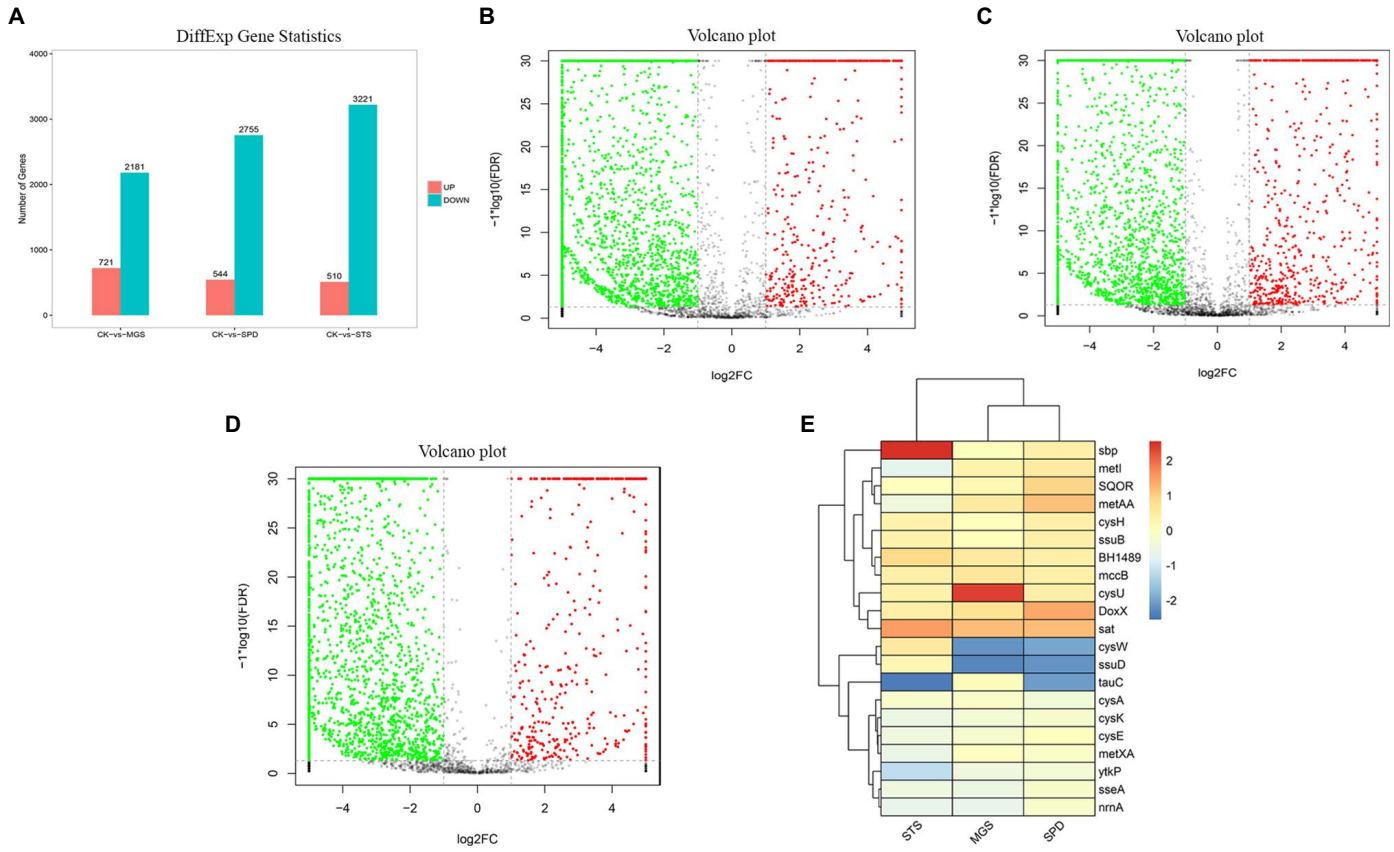
**(A)** Differential gene expression the volcano plot presentation of overall scattered differentially expressed transcripts of NR1 were the black dots; the normal gene (red) showed a significantly and highly expressed gene, whereas the green showed significantly and lowly expressed genes in **(B)** CK versus MGS, **(C)** CK versus SPD, **(D)** CK versus STS, and **(E)** DEGs of genes with high and low expression levels (represented by different colors).

NR1 DNA expression was inhibited by the downregulation of functional genes in sulfur environments. Genome data from transcriptomes of three sulfur groups were used to investigate the role of sulfur metabolism genes which were significantly downregulated (Figure 4).

Most of the downregulated genes in the MGS, STS, and SPD groups were included in all catalytic processes in NR1. *CysPUWA* was initially carried into the cell, and the enzymes catalyzed the reduction of sulfate to sulfite; *cysH* operon encoded a (*PAPS*) reductase. Subsequently, extracellular sulfate was converted to adenylyl sulfate. In the MGS, STS, SPD groups, the downregulated *nrnA* gene converted adenylyl sulfate to 3′-phosphoadenylyl sulfate. The reversible reaction is controlled by *cysC* upregulated in STS. The downregulated gene *TST*, *MPST*, and *sseA* groups controlled the conversion of thiosulfate in SPD, STS, and MGS. Similarly, methanesulfonic acid uses *ssuD* and *msuD* in the conversion of sulfite. Alkenesulfonate was converted to sulfite with the help of *ssuD* enzymes even with the *cysH* operon, which was activated by sulfur deficiency and suppressed by cysteine. When cells are grown in the presence of sulfate or sulfite, the *cysJ/I* operon is transcribed at a higher level and converted to hydrogen sulfide.

**Figure 5:**
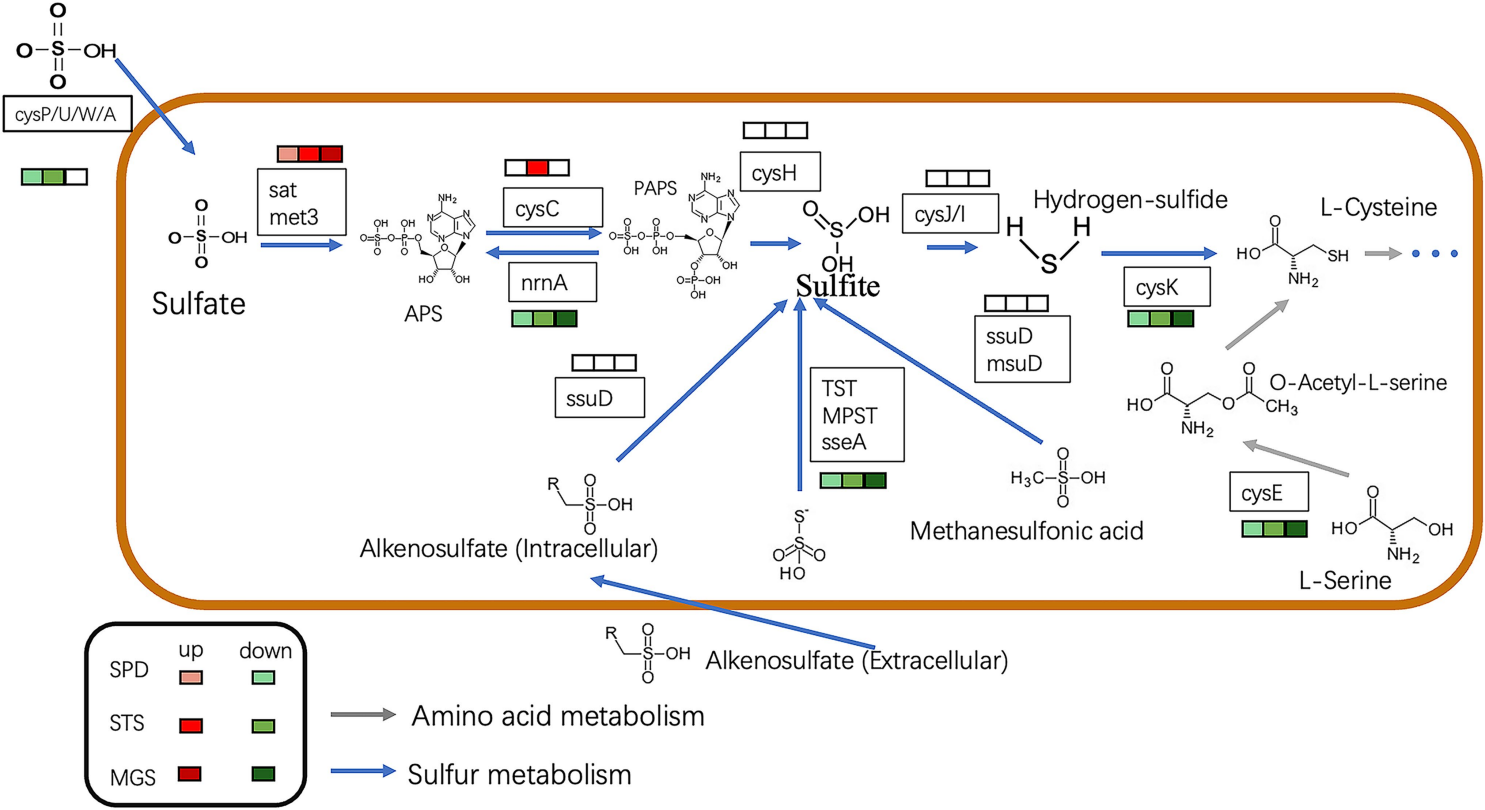
The picture shows the expression of the sulfur metabolism pathway in different transcription component groups in NR1. The different colored squares under the gene name represent the differences in different groups. The green type is down-regulated, and the red type is up-regulated. The blue arrow indicates sulfur metabolism, whereas the grey arrows show amino acid metabolism.

These results suggest that *CysK* could also have a down regulatory function in SPD, STS, and MGS groups that control L-cysteine synthesis. Similarly, *cysE* gene converted O-Acetyl-L-serine into L-cysteine. Even though that the *cysH* operon is activated by sulfur deficiency and suppressed by cysteine, *CysK* catalyzes the final step in such pathways (Figure 5).

### Real-Time Quantitative PCR Analysis

**Figure 6:**
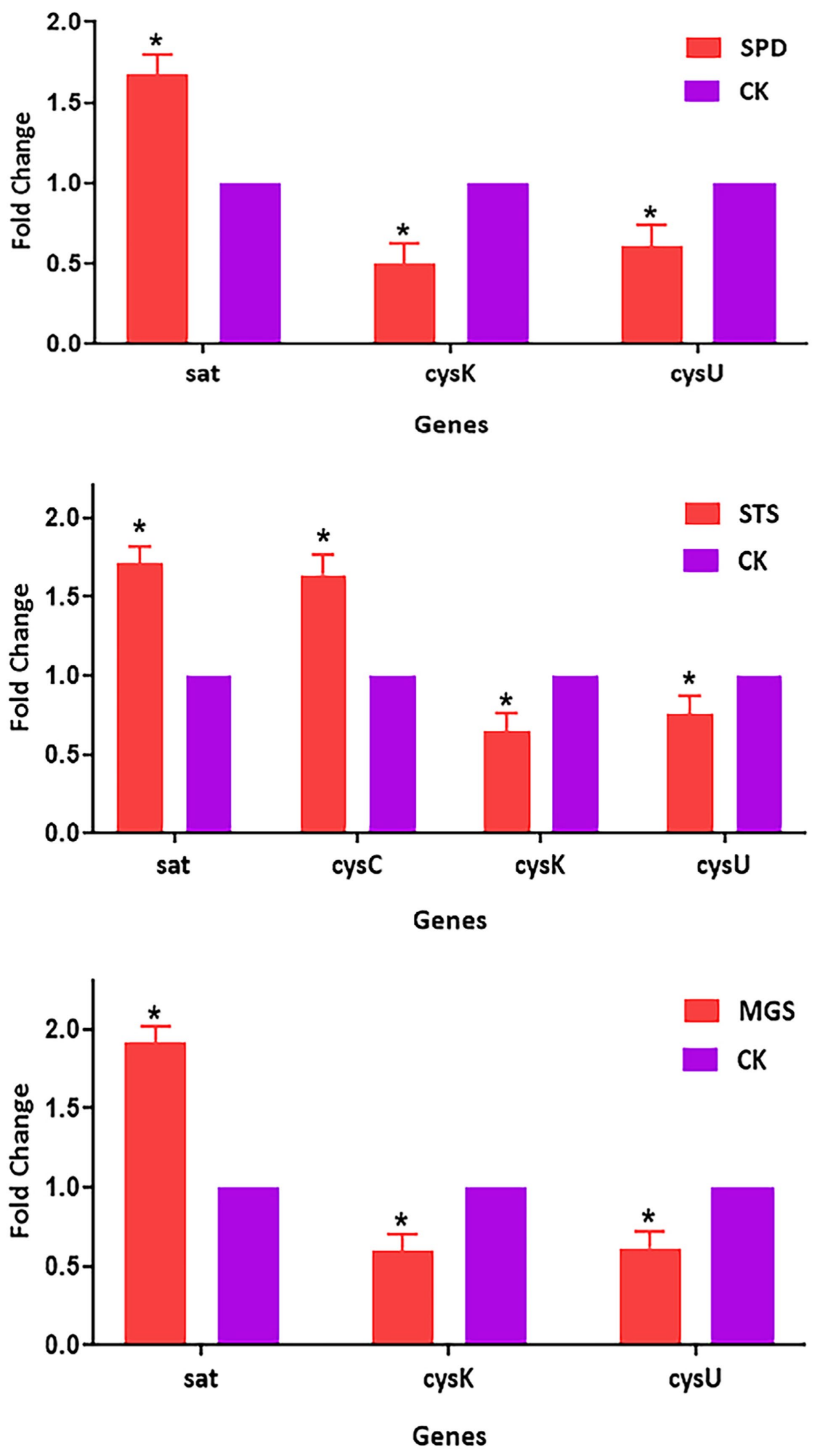
Verification of relative gene expression levels of sat, *cysC, cysK*, and *csyU* by using real-time *qPCR* analysis. The data are presented as mean ± SD, and the statistical significance determined was *p* < 0.05. The expression of the β-actin was used as a reference gene. An asterisk (*) indicates a statistically significant difference (*p* < 0.05) between the groups of sulfur powder (SPD), sodium thiosulfate (STS), magnesium sulfate (MGS), and control (CK).

The transcriptomic data of four sulfur metabolism genes were validated by real-time quantitative PCR analysis. The real-time quantitative PCR showed that gene expressions had similar arrays. The validation of the transcriptomic data was performed by analyzing the expression patterns of *sat, cysC*, *cysK*, and *csyU* by using real-time qPCR. The expression levels of *sat* and *cysC* were upregulated in SPD, STS, whereas *cysK* and *csyU* were downregulated in SPD, STS, and MGS. The results of real-time quantitative PCR were consistent with the transcriptome data (Figure 6).

## Discussion

Sulfur is an essential nutrient for mangrove plant growth and development, and plants only absorb it in the oxidized state ( ${\mathrm{SO}}_{4}^{2-}$ ; Ivanova et al., 2003). Sulfur also exists as a reducing agent, such as sulfide, and is harmful to aquatic animals (Friedrich et al., 2001). Therefore, our study provides investigation on the sulfur-oxidizing bacteria group in mangrove sediments to elucidate mangrove rehabilitation and ecologic treatment through bacterial characterization, sulfur source utilization, and metabolic pathway. Furthermore, we also investigated their genetic behavior through whole-genome and transcriptome sequencing.

Since the introduction of high-throughput DNA sequencing technologies and their applications, the importance of 16S rRNA sequence analysis for bacterial identification and 16S rRNA sequence analysis has become extremely important. A comparative study of the 16S rRNA gene sequence revealed 99% identical to *B. cereus* (Hakovirta et al., 2016). Our study found that NR1 has more than 99% 16S rRNA gene similarity to *B. cereus* in the same clade.

Plant growth-promoting rhizobacteria play a significant role in promoting health and an intentional higher level of tolerance to various significant biotic and abiotic stresses. Vidic et al. (2020) reported that *B. cereus* was Gram-positive and rod-shaped and had an off-white colony. Our work revealed that the cells are Gram-positive, spore-forming, and motile rods with milky white colonies. The findings of this study were consistent with those of Vidic et al. (2020). *B. cereus* was screened to produce enzymes, lipase, protease, phytase, and amylase. We evaluated eight different enzymatic media in this study. The phytase, insulinase, xylanase, protease, potassium, and ammonia nitrogen media showed negative growth activity. By contrast, the glycosidase medium showed strong enzyme synthesis, and the phosphorous medium showed weak interactions (Latorre et al., 2016). Moreover, we demonstrated that NR1 was susceptible to all antibiotics with positive oxidase and catalase reactions. We observed that the NR1 optimum Ph., growth temperature, and salt concentration were pH 7, 17 h, and 4%, respectively. Among all nitrogen sources, D-Arginine was highly utilized by NR1. Among carbon sources, only sucrose was highly utilized.

Dibenzothiophene (DBT) and its derivatives are widely known as model compounds in BDS studies, and a variety of DBT-desulfurizing microorganisms, such as the *Rhodococcus*, have been isolated (Raheb et al., 2009). Ohshiro et al. (2005) and Raheb et al. (2009) reported that monooxygenases (*DszA* and *DszC*), a desulfinase (*DszB*), and an oxidoreductase (*DszC*) were involved in the 4 s pathway (*DszD*). The *dszC* gene had been shown to catalyze the stepwise sulfur oxidation of DBT to DBT sulfone *via* DBT 5-oxide (DBTO; DBTO_2_). *DszA* converted DBTO_2_ to 2-(2-hydroxyphenyl) benzene (HBP) sulfinate (HBPSi-) and was desulfinated to generate HBP and sulfite by the aromatic sulfinic acid hydroxylation encoded by the *dszB* gene. Moreover, *DszA* and *DszC* are two monooxygenases that require FMNH_2_ from *DszD* (Ohshiro et al., 2005; Kilbane and Robbins, 2007). Our findings showed that NR1 desulfurized DBT *via* the 4S pathway at a 90% of desulfurization rate. Previously reported strains of the T7b can desulfurize 25–59% DBT, including *MG1 consortium* and *Sphingomonas subarctica* (Awadh et al., 2020). Overall, we observed that NR1 is a reflective mangrove bacterium in BDS that can degrade organosulfur compounds found in mangrove ecosystems.

Whole-genome sequencing has largely been used as a research tool (Wortmann et al., 2015). In the present study, we demonstrated that NR1 whole genome is related to *B. cereus*. A previous study reported that a *B. cereus* ATCC 14579 genome sequence had a total assembled genome that was 5,426,909 bp long; DNA contigs (plasmids) were 2 (1), and coding sequence (bp) was 4,559,996 (Ivanova et al., 2003). The RNA operons were 13 with a GC content of 35.3%, and the total CDSs were 5,366 (Ivanova et al., 2003). Our study presented that the NR1 whole genome (5,420,664 bp) had a genome size of approximately 8.11 Mb and a GC content of about 35.62%. In this study, a total of 5,305 functional genes were predicted for NR1, among which 42 rRNA were observed, 107 were related to tRNAs, 125 were related to ncRNAs, 2 contigs, and 33 repeat_region. Furthermore, 1,436 genes were annotated in the functional KEGG database, 15,855 genes were in the GO database, and 4,515 genes were in the Nr database.

A total of 11–15 million clean reads from three groups (SPD, MGS, and STS) with high GC contents were used to assemble the transcriptome data. Our experimental findings showed that gene expression revealed 5,305 genes in each group. KEGG annotations were associated with sulfur metabolism, nitrogen metabolism, photosynthesis, valine, leucine, and isoleucine degradation (Supplementary Figure S16). Moreover, GO was related to biological process, cellular components, and molecular functions (Supplementary Figure S15).

Sulfur is an essential macronutrient for all organisms. It can be found in the amino acids cysteine and methionine and a variety of cofactors and prosthetic groups, such as FeS centers, thiamine, and a variety of biologically active compounds (Bykowski et al., 2002). Mangrove plants can absorb inorganic sulfate from the soil, convert it to sulfide, and integrate it into bioorganic compounds (Vauclare et al., 2002). Plant growth may be restricted due to harsh environmental conditions, and the reduction of sulfate assimilation prevents the accumulation of reduced sulfur compounds, thereby decreasing the cellular environment. Sulfur oxidation bacteria use aerobic oxidation to convert elemental sulfur to sulfuric acid. *Acidithiobacillus ferrooxidans* and *Thiobacillus thioparus* can oxidize sulfur to sulfite using an oxygenase enzyme. However, it is assumed that an oxidoreductase and an energy-saving mechanism could be used (Friedrich et al., 2001).

Sulfur incorporation into organic substances is accomplished by producing L-cysteine from inorganic sulfate. In the absence of inorganic sulfate, techniques for producing L-cysteine from organic compounds can be used. Sulfate is the biosphere’s most prevalent source of sulfur. Plants can absorb inorganic sulfate from the soil, converting it to sulfide and integrating it into bioorganic molecules. Sulfate is first activated by ATP sulfurylase (ATPS) to adenosine 5-phosphosulfate in the sulfate assimilation pathway (APS; Tavares and Amâncio, 2017).

The current study identified 36 and 17 sulfur metabolism-related genes using whole-genome and transcriptome data, respectively. These genes are required for assimilatory sulfate reduction. The genetic composition of the sulfate/thiosulfate transport system consists of cysP, cysU, cysW, and cysA and can generate thiocyanate and amino acid metabolism through cysteine biosynthesis SPD, MGS, and STS. *APS* and *PAPS* are reduced to sulfite and is catalyzed to sulfide by sulfite reductase (Albanesi et al., 2005). The results were confirmed by analyzing plants with modulated *APS* reductase expression; precise accumulation of reduced sulfur compounds was found in plants overexpressing APS reductase (Koprivova and Kopriva, 2014). The *cysK* mutation was also correlated with *nrnA e*xpression, which controls the synthesis of L-cysteine. Many reports indicated that H_2_S is a signal in plants; it protects them against a broad range of stresses and promotes growth under certain conditions (Lisjak et al., 2013; Kushkevych et al., 2020).

The biosynthesis of cysteine is divided into two phases, i.e., (1) formation of O-acetylserine is the first step in the cells and (2) the enzyme serine acetyltransferase catalyzes the reaction. The second phase reaction involves the participation of O-acetylserine-(thiol)-lyase in the formation of cysteine from sulfide and O-acetylserine. These enzymes merge to produce a cysteine synthase encoded by the gene *CysK.* Our study is in line with Kushkevych et al. (Kredich, 2008), who reported that APS is the metabolite of a transcription factor (*CysC*), which phosphorylates it using a second ATP molecule. The sulfite is reduced by NADPH-sulfite reductase, a two-subunit enzyme encoded by the c*ysJIH* operon (Kredich, 2008). Alkenesulfonate is converted to sulfite and represses *ssu* enzymes in sulfate media.

## Conclusion

The main conclusion from our research is that the biodesulfurization of bacteria from mangrove sediments increases the genomic understanding of the study. NR1 was a *B. cereus* strain with a sequence length of 5,420,664 bp and two contigs. Additionally, 36 genes of the NR1 strain and 5,305 genes in the whole-genome sequences were directly implicated in sulfur metabolism. The GO database was used to annotate 15,885 genes. Around 4,390 (27.68%) of the genes were associated with molecular functions; 35,168 (21.58%) with cell components; and 7,947 (50.12%) with biological processes. The Nr database annotated 4,551 (63.84%) of the NR1 genes in which 1,436 genes were annotated by the KEGG database. We initially demonstrated 90% DBT degradation activity on NR1. This was indicated by the sulfur metabolism of assimilation sulfate reduction-related genes, such as the *cys, APS*, *PAPS*, *ssu*, and *TST* gene families.

Moreover, bacteria grown in sulfur media exhibited downregulated *cysK* genetic variants correlated with *nrnA* expression, controlling L-cysteine synthesis. This finding demonstrated a scientific basis for future studies and application to mangrove rehabilitation and ecological treatment by examining bacterial characterization and the metabolic pathway for sulfur degradation.

## Data Availability Statement

The datasets presented in this study can be found in online repositories. The names of the repository/repositories and accession number(s) can be found online at: https://www.ncbi.nlm.nih.gov/genbank/, CP090421- CP090422; https://www.ncbi.nlm.nih.gov/, SRR17081772-SRR17081783.

## Author Contributions

CJ, BY, and HT: conceptualization. CJ and HT: resources. MK and YS: data curation. MK, YS, ZL, and SS: methodology and software. CJ: supervision. MK: writing original draft preparation. MK, YS, SK, MH, HT, BY, and CJ: writing review and editing. All authors have read and agreed to the published version of the manuscript.

## Funding

This research was supported by the National Natural Science Foundation of China (grant no. 31760437), the Funding Project of Chinese Central Government Guiding to the Guangxi Local Science and Technology Development (grant no. GUIKEZY21195021), the Natural Science Fund for Distinguished Young Scholars of Guangxi Zhuang Autonomous Region of China (grant no. 2019GXNSFFA245011), the China-ASEAN International Innovative Center for Health Industry of Traditional Chinese Medicine (grant no. AD20297142), and the Innovation Project of Guangxi Graduate Education (grant no. YCBZ2021012 and YCSW2021064).

## Conflict of Interest

The authors declare that the research was conducted without any commercial or financial relationships that could be construed as a potential conflict of interest.

## Publisher’s Note

All claims expressed in this article are solely those of the authors and do not necessarily represent those of their affiliated organizations, or those of the publisher, the editors and the reviewers. Any product that may be evaluated in this article, or claim that may be made by its manufacturer, is not guaranteed or endorsed by the publisher.

## Footnotes

^1^ https://www.bgi.com/

^2^ https://www.ncbi.nlm.nih.gov/

^3^ http://www.genome.jp/tools/kaas/

## Supplementary Material

The Supplementary Material for this article can be found online at: https://www.frontiersin.org/articles/10.3389/fmicb. 2022.856092/full#supplementary-material
