# Supplementary material for: Whole-Genome and Transcriptome Sequencing-Based Characterization of Bacillus Cereus NR1 From Subtropical Marine Mangrove and Its Potential Role in Sulfur Metabolism
Source: Front Microbiol. 2022 Mar 9;13:856092. doi: 10.3389/fmicb.2022.856092 (PMC8959591; doi:10.3389/fmicb.2022.856092)
Supplement: Supplementary file 1 [file Table_1.DOCX]

Supplementary Material

Whole genome and transcriptome sequencing based characterization of *Bacillus cereus* NR1 from subtropical marine mangrove and its potential role in sulfur metabolism

**Muhammad Kashif^1^, Zhaomei Lu^1, 6^, Yimeng Sang^1^, Bing Yan^2^, Sayed Jalil Shah^3^, Sohail Khan^1^, Muhammad Azhar Hussain^4^, Hongzhen Tang^5,^ * and Chengjian Jiang ^1, 2,^ ****

^1^ State Key Laboratory for Conservation and Utilization of Subtropical Agro-bioresources, Guangxi Research Center for Microbial and Enzyme Engineering Technology, College of Life Science and Technology, Guangxi University, Nanning 530004, China.

^2^ Guangxi Key Lab of Mangrove Conservation and Utilization, Guangxi Mangrove Research Center, Guangxi Academy of Sciences, Beihai 536000, China.

^3^ MOE Key Laboratory of New Processing Technology for Non-ferrous Metals and Materials, Guangxi Key Laboratory of Processing for Non-ferrous Metals and Featured Materials, School of Chemistry and Chemical Engineering, Guangxi University, Nanning 530004, China.

^4^ Alfa Diagnostic Services, Faisalabad, Pakistan.

^5^ Key Laboratory and Cultivation Base of Prevention and Treatment of Traditional Chinese Medicine on Obesity, Guangxi University of Chinese Medicine, Nanning 530200, China.

^6^ Key Laboratory of Bio-resources and Eco-environment of the Ministry of Education, College of Life Sciences, Sichuan University, Chengdu 610064, China.

*and **Corresponding author

Tel: +86-771-3239403; Fax: +86-771-3270736

Email ID: **: jiangcj0520@vip.163.com (CJ); *: 383744825@qq.com (HZ)


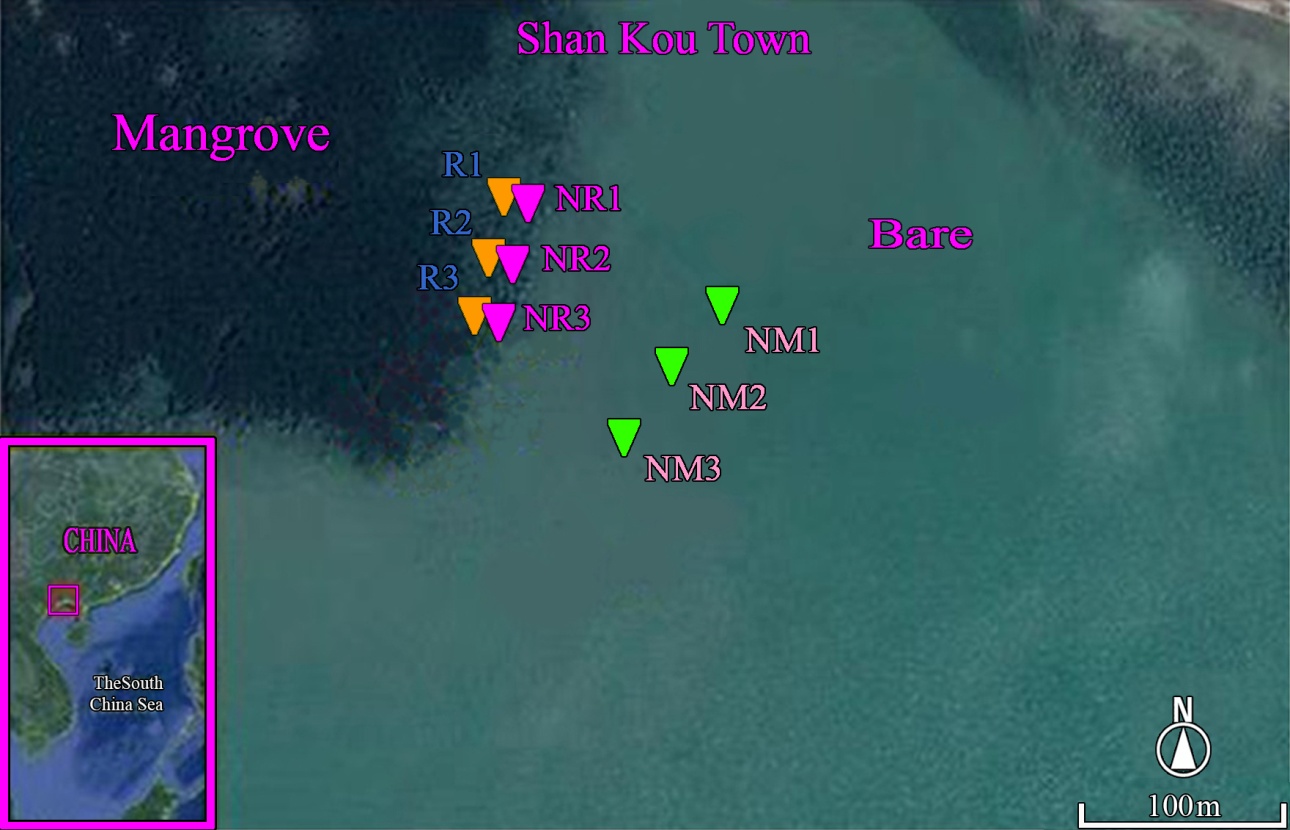


**Fig. S1**. Sample sites collection and distribution are listed. (The sampling sites for the non-mangrove area are designated by the green labels NM1, NM2, and NM3. Orange labels R1, R2, and R3, indicate sampling sites in the rhizosphere area, while pink labels NR1, NR2, and NR3, indicate sampling sites in the non-rhizosphere area from near-root (within 3 cm of the rhizosphere roots).

| **a**  **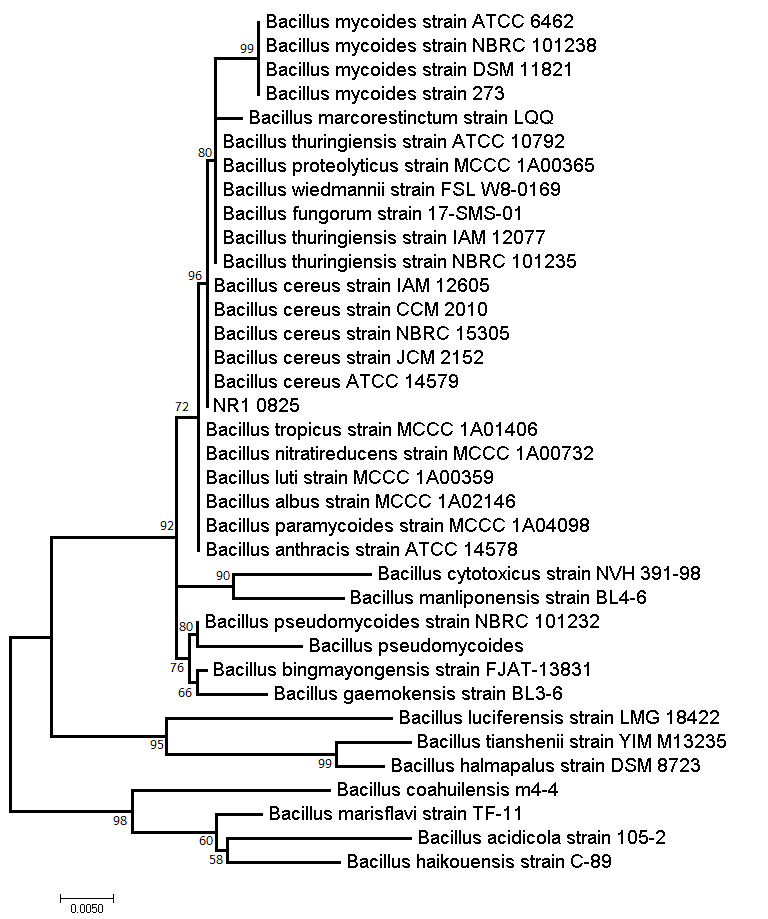** |
| --- |
| **b**  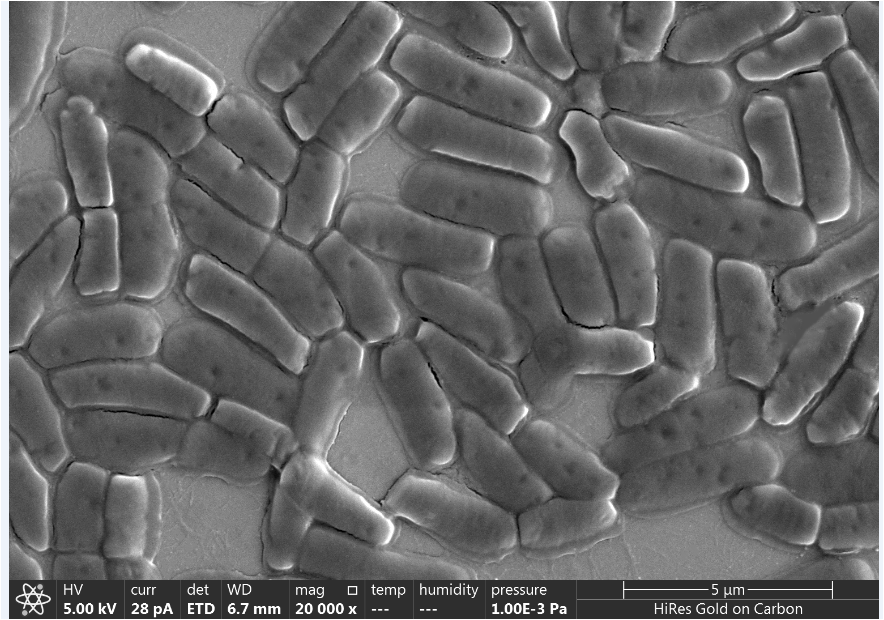 |

**Fig. S2.** (**a)** Phylogenetic relationships of strain NR1 with related species using 16S rRNA gene sequences. **(b)** Scanning electron microscopy (SEM) of strain NR1.


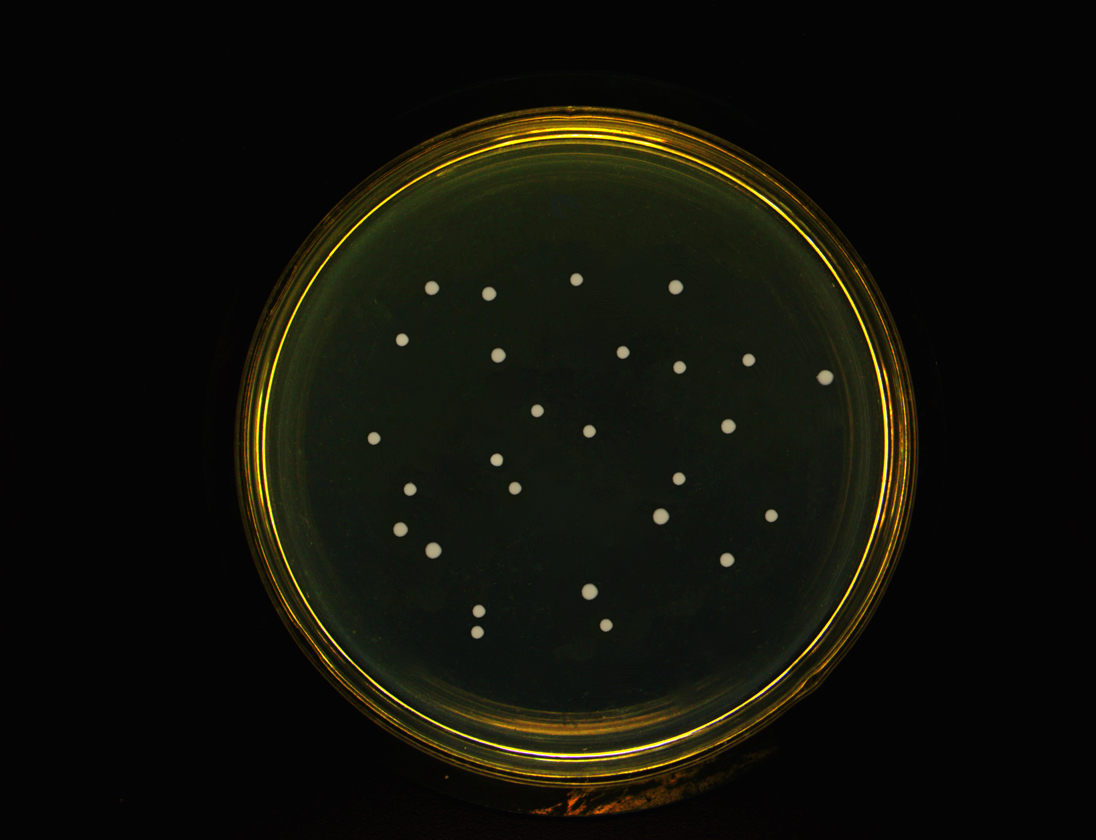


**Fig. S3**. Growth of NR1 colonies observes on Luria–Bertani medium.

**Fig. S4.** Optimal Growth of NR1 at various pH levels.

**Fig. S5.** For NR1, a growth curve was shown at 17 hours on an optimum temperature array.

**Fig. S6.** NR1 growth on 2216E Marine medium at temperatures ranging from 20 to 60°C.

**Fig. S7.** Effect of varying sodium chloride concentration with the interval of 1% (w/v) from 0-16%.


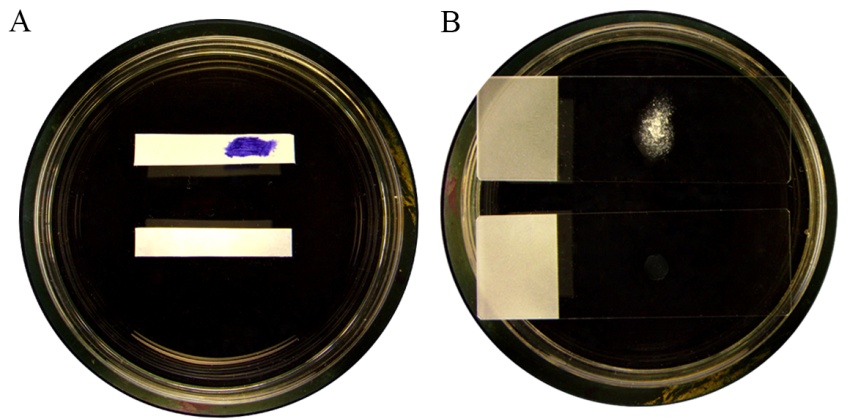


**Fig. S8**. (**A)** Tetramethyl-p-phenylenediamine dihydrochloride, oxidase 1% (v/v), and **(B)** catalase tests with an average of 3% hydrogen peroxide, respectively, are used in the tests.


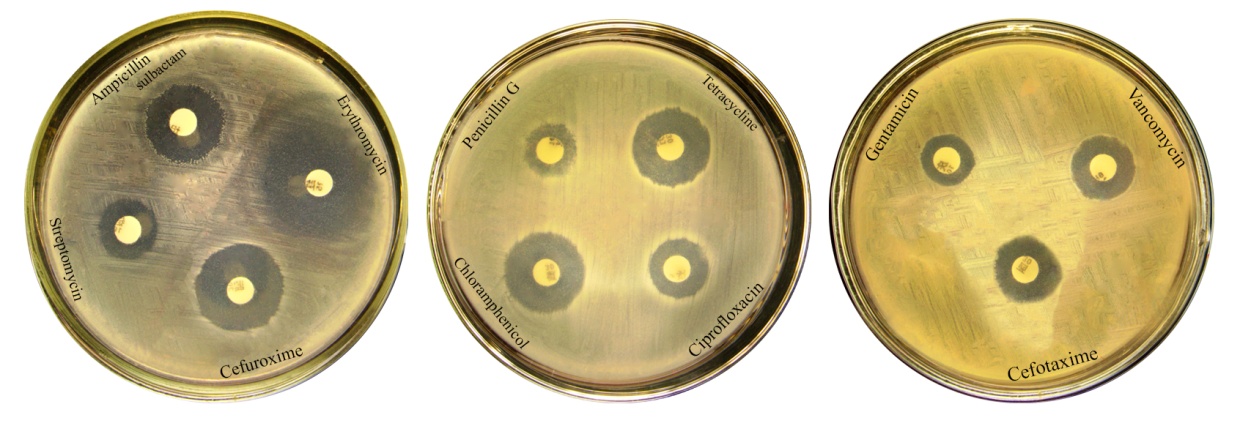


**Fig. S9**.  NR1 antibiotic susceptibility plates demonstrate zones of inhibition for various antibiotic discs.

**Fig. S10.** The growth of NR1 in various nitrogen sources.

**Fig. S11.** The growth of NR1 in various carbon sources.


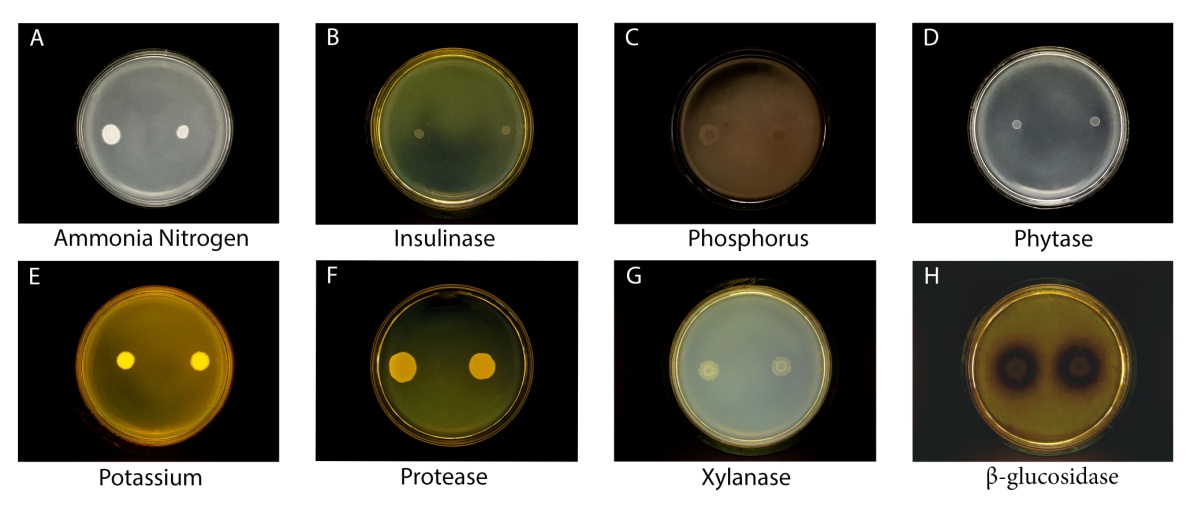


**Fig. S12.** NR1 exhibits an array of enzymatic production activities on a variety of corresponding enzyme mediums.





**Fig. S13.** The 4S pathway converts DBT to 2-HBP and sulfite by the activities of two monooxygenases (*DszC*, *DszA*), a desulfurase (*DszB*), and an oxidoreductase (*DszD*). *DszC* sequentially oxidizes DBT to generate DBTO and DBTO_2_, _DszA_ catalyzes the oxidative C-S bond cleavage in DBTO_2_ to form HBPS, and *DszB* catalyzes the conversion of HBPS to 2-HBP and sulfite (HSO_3_) *DszD* provides the reducing comparable (FMNH_2_) essential for the function of *DszC* and *DszA.*

| **a**  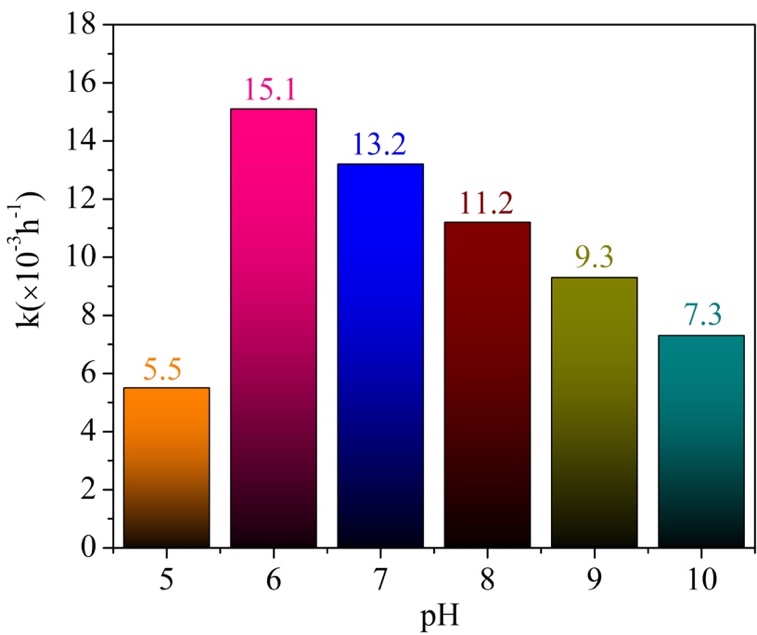 | **b**  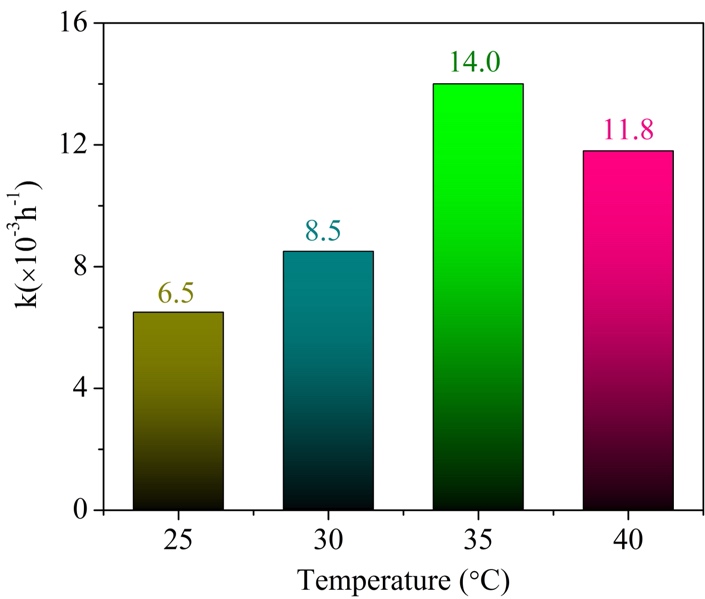 |
| --- | --- |
| **c**  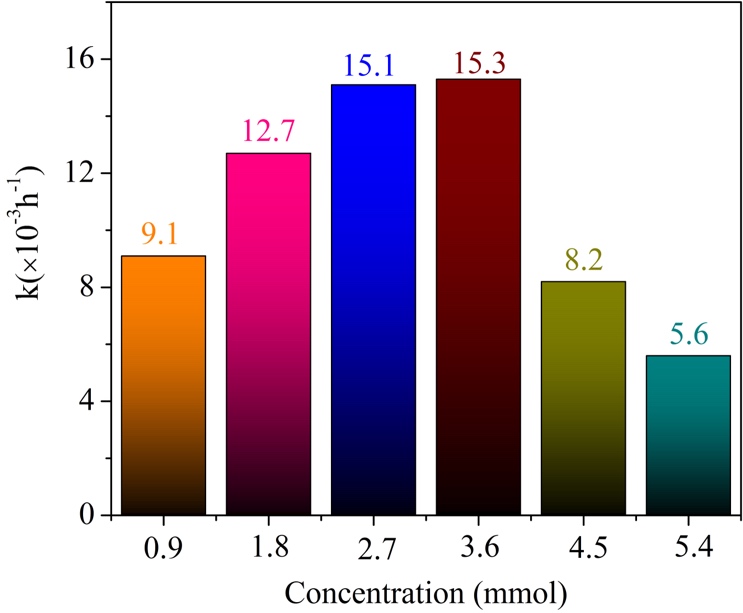 | |

**Fig. S14**. Pseudo-first order apparent rate constants ((k, h^-1^) of DBT biodegradation by NR1 as a consequence of (a) pH, (b) temperature, and (c) DBT initial concentration.

**Fig. S15.** NR1, GO analysis showing different sulfur genes in various subcellular components.


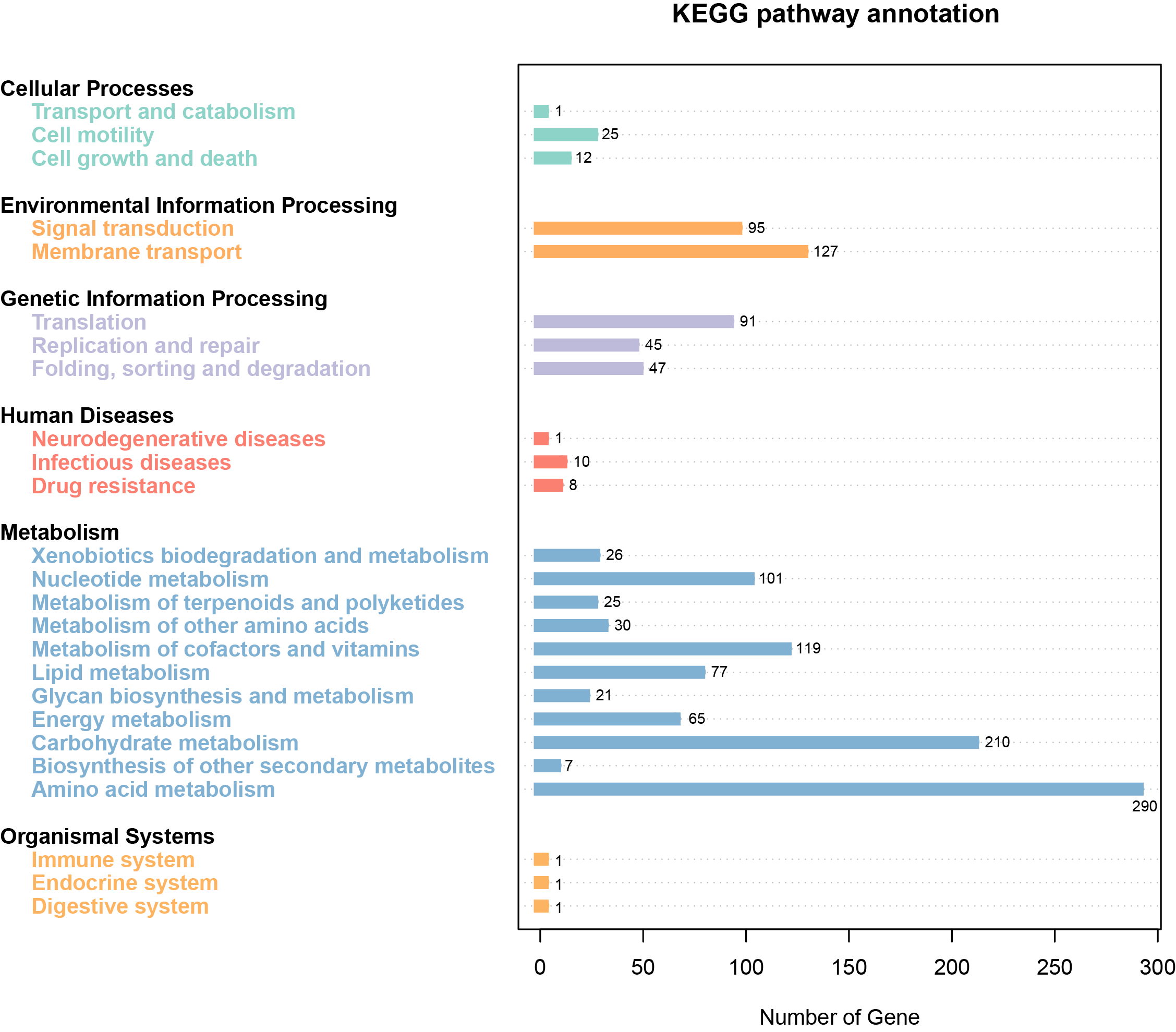


**Fig. S16.** Annotation of KEGG pathways demonstrating the various gene functions of NR1.


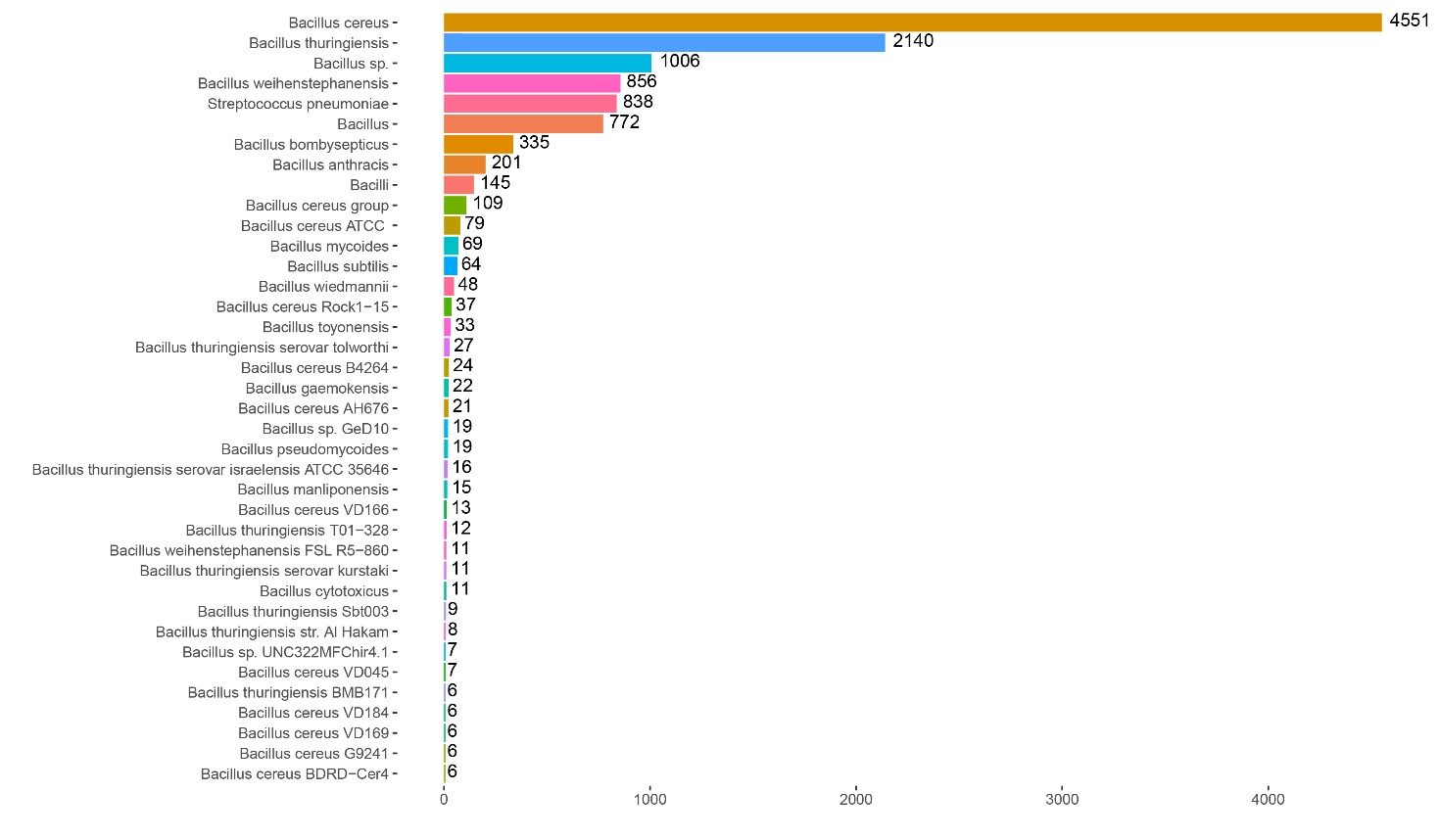


**Fig. S17.** Nr Annotation results signify NR1 comparable genes with other bacterial strains.

**Table S1.** NR1 genomic data statistics.

| **Genomic Data** | **Strains** | |
| --- | --- | --- |
|  |  | NR1 |
| contigs |  | 2 |
| bases |  | 5, 420, 664 bp |
| CDS |  | 5305 |
| rRNA |  | 42 |
| repeat_region |  | 33 |
| tRNA |  | 107 |
| tmRNA |  | 1 |

**Table S2.** The statistics of the sequenced data.

| Sample | Total_Reads | Unmapped_Reads | Mapping_Ratio | Clean Data(bp | Clean Reads Num(%) | GC contents | Q30(%) | Multiple_Mapped_reads |
| --- | --- | --- | --- | --- | --- | --- | --- | --- |
| CK-1 | 7896140 | 403714(5.11%) | 94.89% | 1242631136 | 8494864 (99.93%) | 527576583 (42.46%) | 1157175891 (93.12%) | 173192(2.19%) |
| CK-2 | 8578196 | 413073(4.82%) | 95.18% | 1355547653 | 9260472 (99.92%) | 573266005 (42.29%) | 1261682044 (93.08%) | 202534(2.36%) |
| CK-3 | 9073618 | 462918(5.10%) | 94.90% | 1421972609 | 9707876 (99.92%) | 600417075 (42.22%) | 1318486686 (92.72% | 192354(2.12%) |
| MGS-1 | 9429764 | 426553(4.52%) | 95.48% | 1410425023 | 9617652 (99.91%) | 626571442 (44.42%) | 1315200790 (93.25%) | 51698(0.55%) |
| MGS-2 | 10118224 | 428846(4.24%) | 95.76% | 1525678120 | 10398098 (99.89%) | 673029727 (44.12%) | 1418493814 (92.97%) | 123453(1.22%) |
| MGS-3 | 8113516 | 357245(4.40%) | 95.60% | 1213698913 | 8280838 (99.9%) | 537776576 (44.31%) | 1124950810 (92.69%) | 63682(0.78%) |
| SPD-1 | 9073234 | 61476(0.68%) | 99.32% | 1378338108 | 9369722 (99.88%) | 647555627(46.98%) | 1273640295 (92.4%) | 100092(1.10%) |
| SPD-2 | 7906068 | 50959(0.64%) | 99.36% | 1206714341 | 8211394 (99.88%) | 566472060 (46.94%) | 1122415224 (93.01%) | 115278(1.46%) |
| SPD-3 | 7625296 | 42018(0.55%) | 99.45% | 1158392988 | 7901816 (99.93%) | 544205933 (46.98%) | 1076833518 (92.96%) | 105748(1.39%) |
| STS-1 | 9522602 | 92644(0.97%) | 99.03% | 1520480881 | 10342584 (99.91%) | 649533895 (42.72%) | 1411970969 (92.86%) | 355152(3.73%) |
| STS-2 | 7735898 | 88081(1.14%) | 98.86% | 1235079747 | 8430832 (99.89%) | 534786064 (43.3%) | 1147707034 (92.93%) | 311025(4.02%) |
| STS-3 | 8849772 | 83328(0.94%) | 99.06% | 1398165832 | 9541548 (99.91%) | 598822336 (42.83%) | 1301965297 (93.12%) | 302274(3.42%) |

**Table S3.** Significant number of DEGs.

| Control | Case | Up-regulated Genes | Down-regulated Genes | Total DEGs |
| --- | --- | --- | --- | --- |
| CK | MGS | 721（24.85%） | 2181（75.15%） | 2902 |
| CK | SPD | 544（16.49%） | 2755（83.51%） | 3299 |
| CK | STS | 510（13.67%） | 3221（86.33%） | 3731 |

|   MGS |   SPD |
| --- | --- |
|   STS | |

**Fig. S18.** The KEGG pathway enrichment bubble diagram of DEGs. As shown in the figure, Richfactor is the ratio of the number of differentially expressed transcripts found in this pathway entering to the total number of transcripts. The higher the Richfactor, the greater the enrichment. Qvalue is the PValue after multiple hypothesis testing and correction, with a value range of 0 to 1. The enrichment will be greater the closer it gets to zero. On the graph, the top 20 pathways are plotted in order of QValue from lower to higher.

**a**


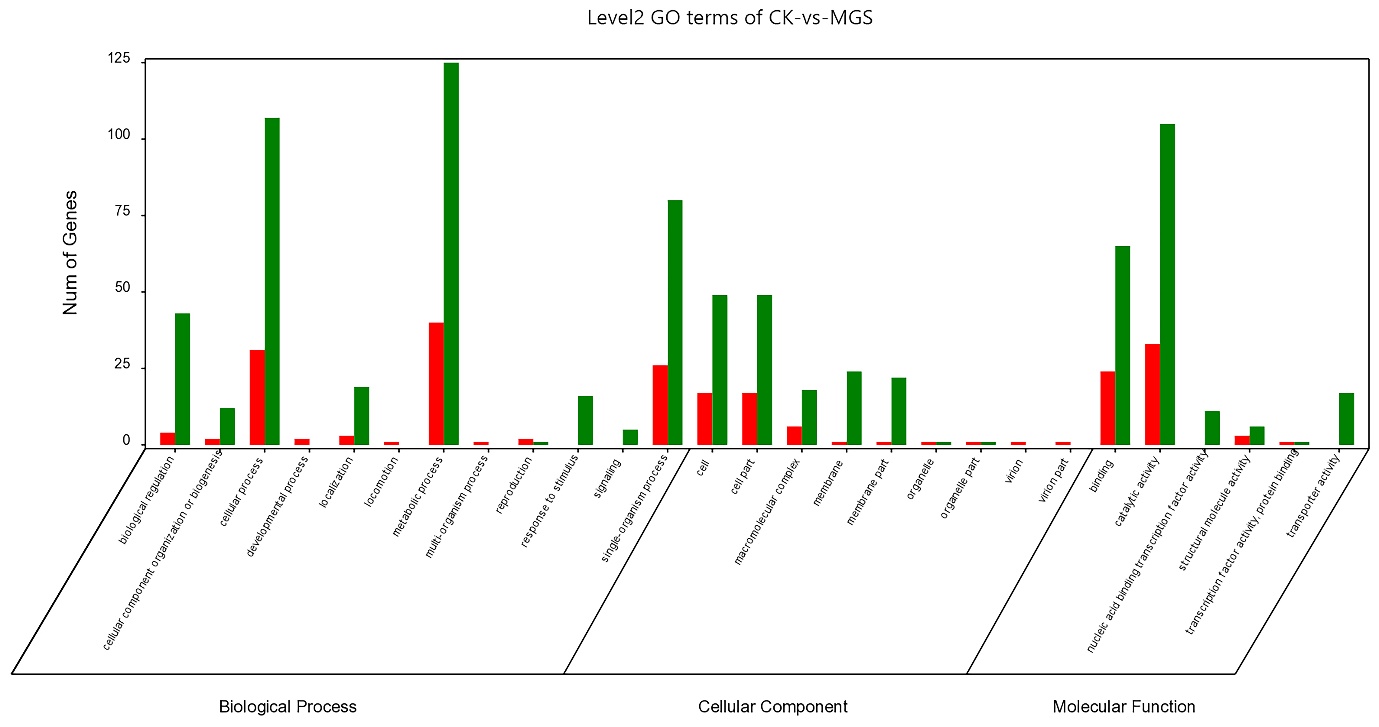


**b**


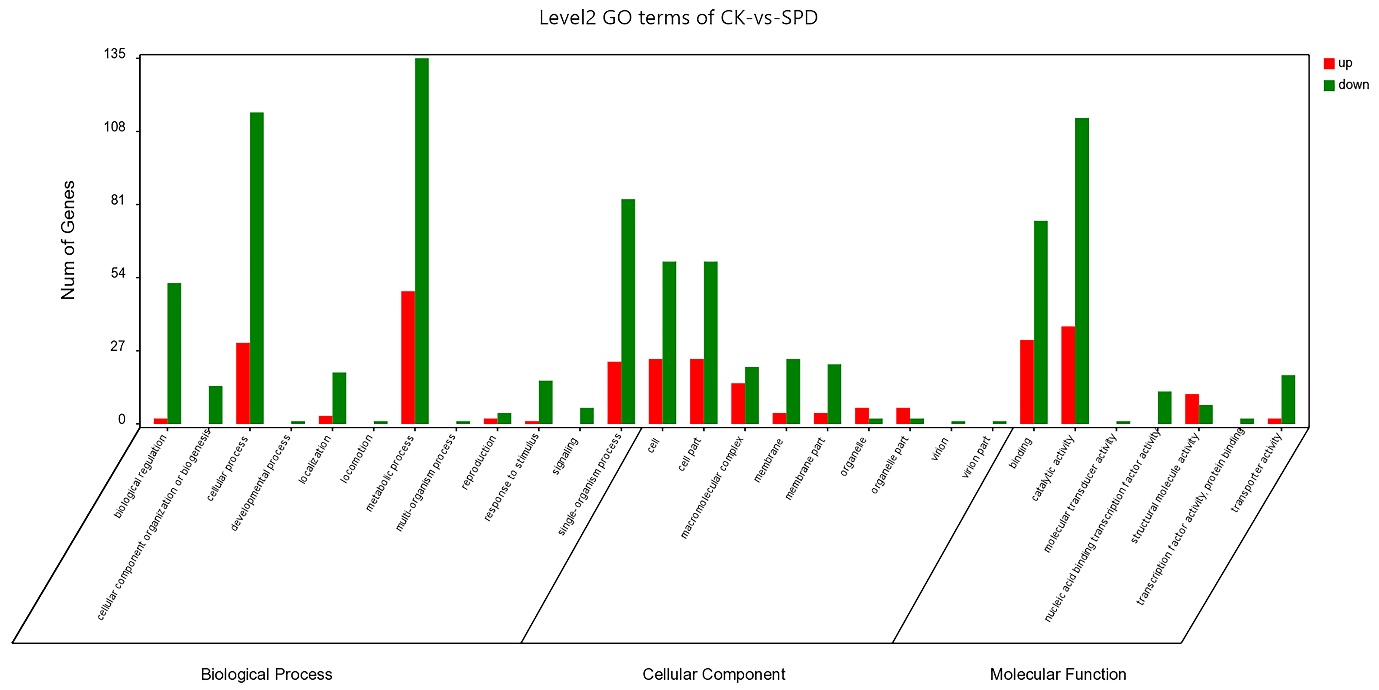


**c**


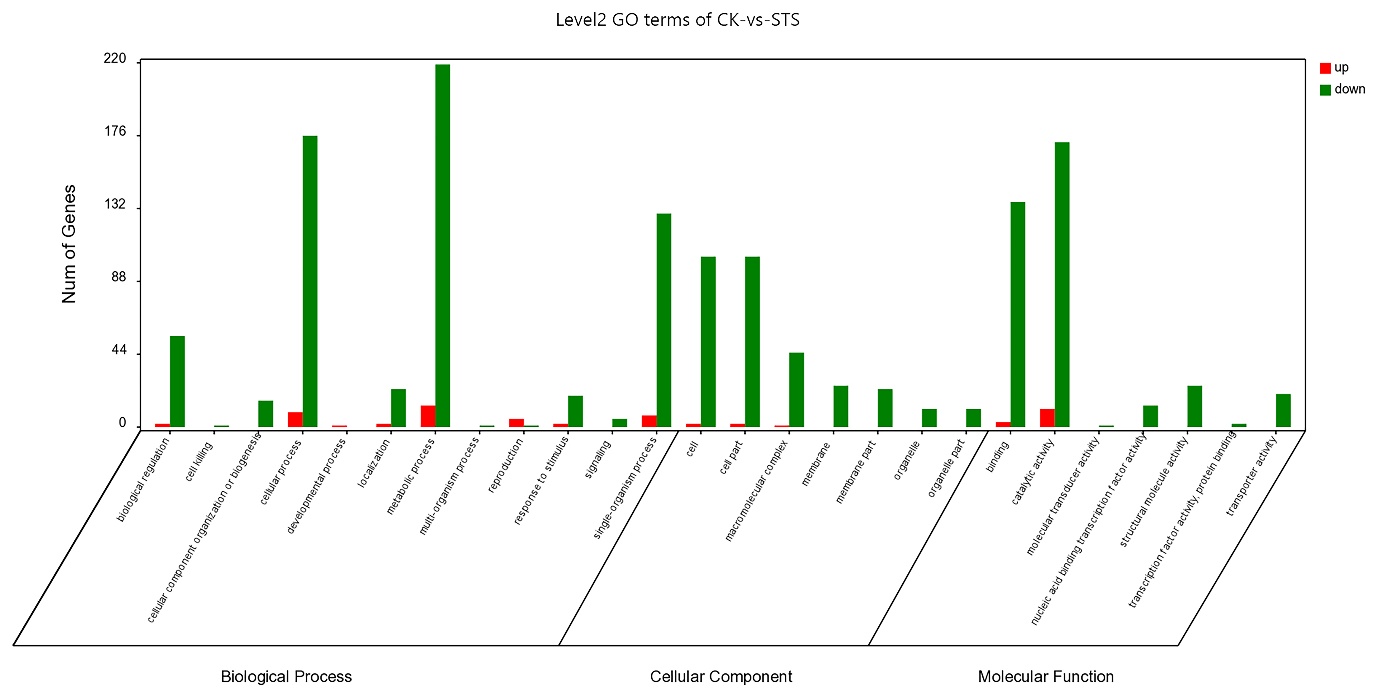


**Fig. S19.** GO classification results of DEGs. (a) CK-vs-MGS, (b) CK-vs-SPD (c) CK-vs-STS.
